# Machine learning-assisted highly efficient thermal management in function-oriented thermochromic smart windows

**DOI:** 10.1038/s41377-026-02369-4

**Published:** 2026-06-22

**Authors:** Zhengui Zhou, Changyuan Chen, Bin Li, Rong Liu, Shouqin Tian, Bin Hu, Yi Long

**Affiliations:** 1 Department of Electronic Engineering, The Chinese University of Hong Kong, Shatin, New Territories, Hong Kong SAR, China; 2 State Key Laboratory of Advanced Glass Materials, Wuhan University of Technology, Wuhan, China; 3 Wuhan National Laboratory for Optoelectronics, School of Optical and Electronic Information, Huazhong University of Science and Technology, Wuhan, China; 4 Shenzhen Huazhong University of Science and Technology Research Institute, Shenzhen, China

**Keywords:** Photonic devices, Nanoparticles

## Abstract

Windows contribute significantly to energy inefficiency in electric vehicles and buildings, and the development of smart windows with customized optical responses catering to various purposes presents a significant challenge. Vanadium dioxide (VO_2_) Fabry-Perot resonator has emerged as an effective device to modulate heat transfer passively to enhance energy-saving performance, with its efficacy highly limited by the complex interrelationship between the structure and multispectral selectivity. This study introduces a physics-guided neural network to design energy-efficient thermochromic smart windows for directional privacy protection (DPP). Guided by machine learning predictions, the VO_2_ nanoparticle size- and spacer-controlled DPP smart window exhibits a targeted luminous transmission (<0.15) with both high modulation of near-infrared transmittance of 0.12 and longwave infrared radiation emissivity of 0.56, surpassing both commercial and the best-reported windows. When applied in electric vehicles and building envelopes, the DPP smart window demonstrated superior thermal management compared to commercial counterparts. This work provides a framework for the inverse design of function-oriented smart windows, advancing energy-efficient solutions for real-world applications.

## Abstract (web summary)

A physics-guided neural network is proposed to inversely design energy-efficient thermochromic windows with directional privacy protection.

## Graphical abstract

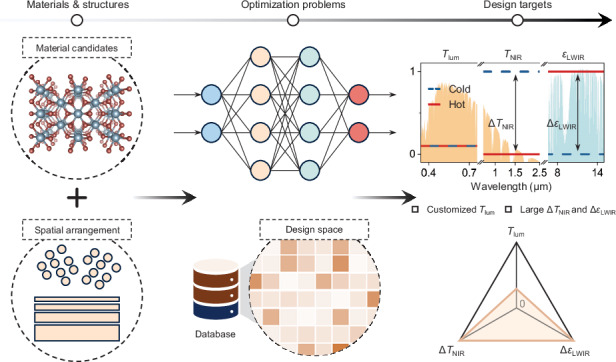

## Introduction

Windows are among the least energy-efficient components in electric vehicles and buildings, accounting for 50 ~ 75% of the thermal load in electric vehicle cabins and ~45% of total energy losses in buildings^1,2^. Enhancing window energy performance has therefore become a focus in the pursuit of low-carbon thermal management and a sustainable environment^3,4^. Recent advances in smart window technologies have enabled dynamic regulation of light and heat transfer, primarily using electro-, thermo-, mechano-, and photo-chromic materials^5,6^, and solar/thermal dual modulation has emerged as a promising approach for all-weather energy savings^7,8^. Thermochromic materials, particularly those based on vanadium dioxide (VO_2_) Fabry-Perot (FP) resonator, have been demonstrated as a cost-effective passive thermal management through tunable emissivity by exploiting the transition of VO_2_ from its monoclinic VO_2_(M) to rutile VO_2_(R) phase (Table S1)^8–11^. However, achieving enhanced modulation in both longwave infrared radiation emissivity (Δ*ε*_LWIR_) and near infrared transmittance (Δ*T*_NIR_) remains a challenge (Table S2)^8,10–12^. Moreover, windows serve different functionalities to meet specific application demands. Commercial energy‑saving static low‑emissivity (low‑e) glass covers a broad range of functionalities (Fig. S1), with luminous transmittance (*T*_lum_) ranging from 0.1 to 0.9, solar transmittance (*T*_sol_) from 0.1 to 0.6, and longwave infrared emissivity (*ε*_LWIR_) <0.5 (Table S3). For example, the privacy‑glazing segment with customized *T*_lum_ is growing with a market size of ~US$4.3 billion in 2024 and projected annual growth of 6.8%^13^. Therefore, maintaining privacy protection while increasing contrast in Δ*ε*_LWIR_ and Δ*T*_NIR_ to maximize energy efficiency is essential for both fundamental research and industry.

Machine learning (ML) has emerged as a transformative tool in materials science, offering powerful capabilities to accelerate the discovery and optimization of functional materials^14–17^. Unlike traditional trial-and-error methods, ML algorithms can efficiently explore high-dimensional parameter spaces, uncovering complex, nonlinear structure-property relationships even with limited experimental data^18,19^. This data-driven approach has already facilitated significant advancements across various fields, including the development of high-performance catalysts^20^, next-generation energy storage systems^21^, and photonic architectures with tailored optical responses^22–24^.

While integrating ML-driven inverse design with passive thermal management strategies, such as radiative cooling, effectively resolves competing performance trade-offs^22,25,26^, extending this framework to smart windows presents a formidable challenge. This difficulty arises primarily from the physical complexity of simultaneously tailoring dynamic optical responses across multiple distinct spectral bands (Table S4)^22–24,27,28^. Overcoming this hurdle demands an approach that navigates these complex trade-offs while strictly ensuring practical feasibility. By incorporating domain-specific knowledge and physical constraints into ML frameworks^29^, it is possible to move beyond basic predictive models toward rational inverse design, paving the way for the development of next-generation smart windows with customized spectral capabilities.

Herein, a physics-guided neural network was developed to assist in the design of a thermochromic FP smart window for directional privacy protection (DPP). The ML results suggest that spacer thickness predominates Δ*ε*_LWIR_ while smaller VO_2_ nanoparticles (NPs) are needed for enhanced Δ*ε*_LWIR_ and Δ*T*_NIR_. Size-controlled VO_2_ NPs were synthesized for the DPP smart window, which demonstrated superior optical performance, achieving a high Δ*T*_NIR_ of 0.12 and Δ*ε*_LWIR_ of 0.56, outperforming both commercial glass and the state of the art^8^. Compared to the static commercial counterpart, the DPP smart window was subsequently applied in an electric vehicle, which can reduce the maximum car air temperature by up to 3 °C in summer and increase 0.9 °C in winter. Building energy-saving simulations further highlighted the significant energy savings of up to 7% in various mid-latitude cities. By establishing a framework for the inverse design of smart windows, this work paves the way for the development of energy-efficient solutions that can be tailored to meet diverse real-world needs.

## Results

### Design principle

**Fig. 1:**
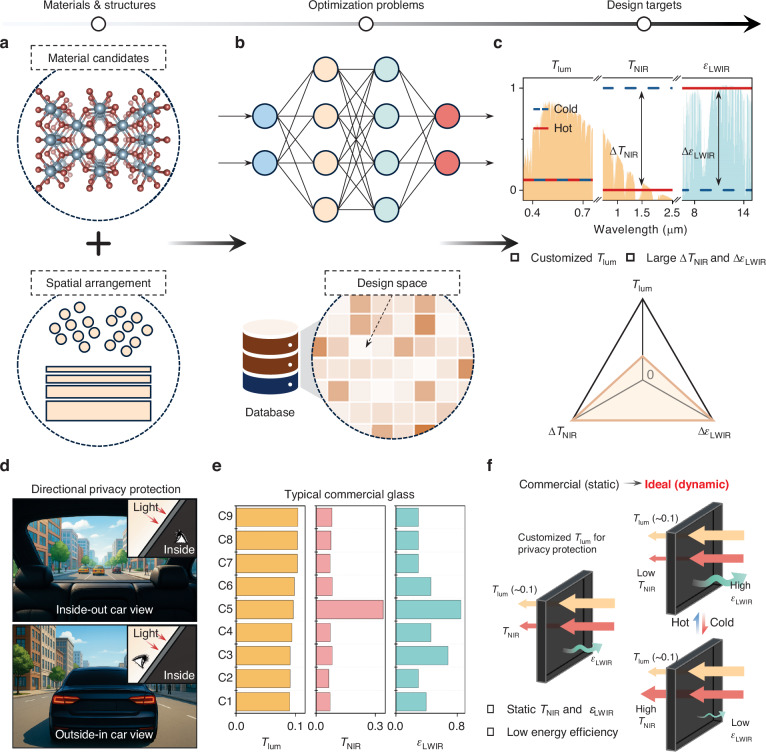
ML-based design process and schematic of the DPP smart window. **a** The proposed ML-based approach, including candidate materials and spatial arrangements. **b** ML-based design with global optimization solutions. **c** ML-based design for customized objective and customer-specific spectral demands. **d** Schematic of the final goal for directional privacy protection. **e** 9 typical commercial glass with statistical *T*_lum_, *T*_NIR_, and ε_LWIR_. **f** Schematic of the proposed DPP smart window compared with commercial glass

We develop an ML-assisted inverse design framework for multilayer photonic structures for an energy-efficient privacy window. As illustrated in Fig. 1a, the design space consists of two primary degrees of freedom: (i) the materials selected, such as thermochromic VO_2_ and other functional layers, and (ii) their spatial arrangement, including the order of layers, their thickness, and structural dimensions. These structural and compositional parameters collectively determine the spectral behavior of the system across the visible, NIR, and LWIR bands. This parameter set is then used as input for a global optimization procedure (Fig. 1b), wherein a physics-guided neural network, trained on computational datasets by finite‑difference time‑domain (FDTD), identifies the optimal configurations within the design space. The ultimate goal is to achieve on-demand inverse design of photonic structures that fulfill user-defined spectral profiles, with the focus on a smart window with a *T*_lum_ of <0.15 for DPP and largely modulated Δ*T*_NIR_ and Δ*ε*_LWIR_, offering significant potential for applications in both vehicles and buildings (Fig. 1c and S2).

The DPP arises from low photopic transmittance (dark tint) combined with exterior-interior luminance asymmetry (Fig. 1d and S3). Under bright outside illumination (*L*_out_ ≫ *L*_in_), the transmitted exterior scene (*T*_lum_·*L*_out_) remains visible to occupants, whereas the transmitted interior luminance (*T*_lum_·*L*_in_) is far below the detection threshold of outside observers adapted to *L*_out_, giving a one-way visual privacy feature. Nine commercial static privacy glasses show a typical *T*_lum_ < 0.15, a *T*_NIR_ spanning 0.06 ~ 0.34, and an *ε*_LWIR_ of 0.29 ~ 0.85 (Fig. 1e, Table S3), incapable of dynamic switching and seasonal adaptivity. In contrast, the DPP smart windows should maintain comparable *T*_lum_ while enabling climate‑adaptive solar/thermal management (Fig. 1f). In hot conditions, it suppresses solar heat gain (low *T*_NIR_) and promotes radiative cooling (high *ε*_LWIR_), whereas in cold conditions it admits more NIR (high *T*_NIR_) and reduces thermal losses (low *ε*_LWIR_). Thus, the DPP smart window would improve the energy-saving potential while preserving the privacy protection function compared with commercial counterparts.

To achieve the aforementioned DPP smart window, a four-layer FP structure was constructed for ML (Fig. S4). From top to bottom, the stack comprises: (i) a VO_2_ NP coating whose optical constants are thermally tuned across the insulator–metal transition^30^ (Fig. S5); (ii) a polymeric spacer was selected for its low loss in the LWIR spectrum (Fig. 6); (iii) a sputtered indium tin oxide (ITO) low‑e layer (Fig. S7); and (iv) a glass substrate.

### ML-based inverse design process

**Fig. 2:**
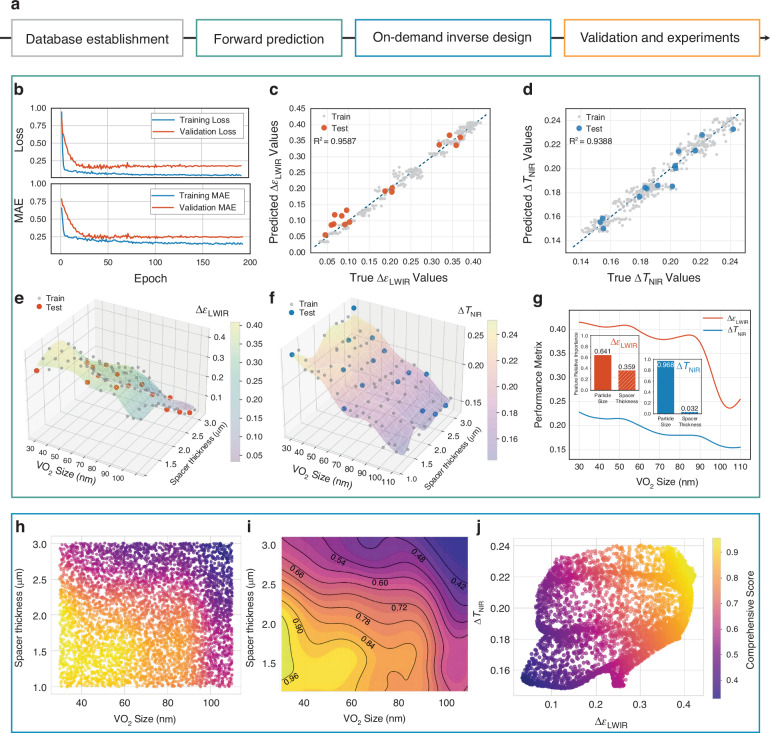
ML-driven inverse design and performance optimization. **a** Workflow of the four-stage ML framework for the inverse design of the VO_2_-based smart window. **b** Training loss and MAE of the physics-guided neural network. **c**, **d** Parity plots comparing FDTD-simulated (ground truth) and ML-predicted values for **c** Δ*ε*_LWIR_ and **d** Δ*T*_NIR_. The strictly isolated test data points align within the augmented training data cloud, demonstrating high predictive accuracy, with R^2^ values of 0.9587 and 0.9388, respectively. **e**, **f** High-fidelity predictive response surfaces illustrating the non-linear dependence of **e** Δ*ε*_LWIR_ and **f** Δ*T*_NIR_ on VO_2_ diameter and spacer thickness across the full design space. **g** Cross-sectional profile of Δ*ε*_LWIR_ and Δ*T*_NIR_ at a fixed spacer thickness of 1.65 µm, with insets showing feature importance analysis for VO_2_ particle size and spacer thickness on (left) Δ*ε*_LWIR_ and (right) Δ*T*_NIR_. **h**, **i** Candidate design space analysis showing **h** the scatter distribution of 5000 generated candidate designs and **i** the corresponding comprehensive performance score contour mapped onto the 2D parameter space. The color mapping in **h**, **i** explicitly represents the comprehensive objective score, where warmer colors indicate candidate designs with great overall performance. **j** Pareto front illustrating the inherent trade-off between Δ*ε*_LWIR_ and Δ*T*_NIR_

The complex and nonlinear relationship between optical performance, particle geometry, and multilayer configuration significantly constrains the design of DPP smart windows, and traditional empirical models fail to account for this intricate interplay. To address this challenge, we propose a comprehensive strategy, outlined in Fig. 2a, which follows a structured four-stage pipeline: (i) database establishment via FDTD simulations, (ii) forward prediction using a physics-guided neural network, (iii) on-demand inverse design to identify optimal configurations, and (iv) validation of the proposed designs. In FDTD modelling, VO_2_ NPs are arranged in a hexagonal close-packed (HCP) configuration as illustrated in Fig. S8, with particle diameters changing from 30 to 110 nm and a spacer thickness ranging from 1.1 to 3.1 μm. The layer of VO_2_ (*n*) is controlled to maintain the thickness of VO_2_ ranging from ~79 nm (three 30 nm sub-layers) to ~127 nm (two 70 nm sub-layers). As detailed in Note S1, coupling the particle size with the layer number is a necessary physical constraint dictated by the transmittance requirement. This ensures the ML optimization remains within valid experimental boundaries rather than generating physically trivial solutions.

A physics-guided neural network^31^ that combines domain knowledge with data-driven learning was developed, which could significantly improve computational efficiency (Note S2, Table S5). To build the computational dataset by FDTD, we systematically sampled 99 structural configurations of the FP multilayer architecture (Figs. S9 and S10, and Table S6), by varying different VO_2_ particle sizes and spacer thickness. To strictly prevent data leakage and rigorously evaluate model generalization, we first isolated a portion of unseen FDTD data points as a strictly hold-out test set. A physics-based augmentation strategy was then employed exclusively on the remaining training data. This augmentation incorporated bivariate spline interpolation to map the continuous physical parameter space, followed by finely tuned structural noise injection. This noise injection was specifically introduced to account for the morphological irregularities of the synthesized VO_2_ NPs compared to the ideal spheres assumed in FDTD simulations. This specific control prevented the artificial smearing of sharp optical resonances, preserving the genuine physical characteristics of the FP interference while expanding the training dataset to ensure comprehensive coverage of the design space. Subsequently, the neural network architecture employs multiple hidden layers, batch normalization, and dropout to ensure robust learning. Crucially, the training process is governed by a physics-guided loss function, utilizing boundary penalties and smoothness regularizations to enforce thermodynamic limits while preserving sharp optical resonance peaks. Consequently, both the total loss and mean absolute error (MAE) decrease steadily and converge without overfitting (Fig. 2b). The model demonstrates high predictive accuracy, achieving *R*^2^ of 0.9587 and 0.9388 for Δ*ε*_LWIR_ and Δ*T*_NIR_, respectively (Fig. 2c, d).

Leveraging this validated forward model, we generated high-fidelity predictive response surfaces across the full design space (Fig. 2e, f), which reveal the complex, non-linear interplay between the structural parameters and optical responses. The optimal modulation occurs at small diameters (<40 nm) and suitable spacer thicknesses. Spacer thickness shows negligible effects on the Δ*T*_NIR_ due to its high broadband transmittance. A cross-sectional profile at fixed spacer thickness of 1.65 μm further confirms this size dependence (Fig. 2g). Additionally, two inset bar charts in Fig. 2g quantitatively assess the relative importance of VO_2_ particle size and spacer thickness for each optical performance metric, focusing on the parameter ranges of 30 ~ 100 nm for particle size and 1.1 ~ 2.1 μm for spacer thickness. These ranges were selected based on their practical relevance and to ensure a representative sampling of the parameter space where significant performance variations were observed in the forward simulations. For Δ*ε*_LWIR_ (left inset), particle size shows a greater influence (0.641) than spacer thickness (0.359). Meanwhile, for Δ*T*_NIR_ (right inset), particle size overwhelmingly dominates, with an importance value of 0.968, far exceeding that of spacer thickness (0.032). These feature importance results quantitatively affirm that small particle size favors both Δ*ε*_LWIR_ and Δ*T*_NIR_, and spacer thickness needs to be tuned for the maximized Δ*ε*_LWIR_.

Building on the validated forward model and its continuous response surfaces, we developed an inverse design framework to navigate the inherent trade-off between Δ*ε*_LWIR_ and Δ*T*_NIR_. By evaluating 5000 physically realizable candidates using a normalized scoring function (Equation 8) with equal weightage of Δ*ε*_LWIR_ and Δ*T*_NIR_, high-scoring designs are sparse and unevenly distributed across the parameter space. To explicitly clarify this performance landscape, the color mapping in the candidate scatter and contour plots (Fig. 2h, i) strictly represents the comprehensive objective score, where warmer colors denote candidate designs with superior overall performance. When mapping the score onto the (VO_2_ diameter and spacer thickness) plane, we identify a well-defined high-performance region centered ~30 ~ 40 nm for VO_2_ diameter and 1.5 ~ 2.0 μm for spacer thickness (Fig. 2i), which precisely aligns with the optimal FP interference condition. Pareto front analysis further quantifies the trade-off between the two objectives (Fig. 2j). Our top-ranked configuration with ~30 nm VO_2_ NPs achieves the maximum for Δ*ε*_LWIR_ and Δ*T*_NIR_, demonstrating a balanced dual-optimality that cannot be achieved through traditional trial-and-error or single-objective design approaches.

### Preparation and characterization

**Fig. 3:**
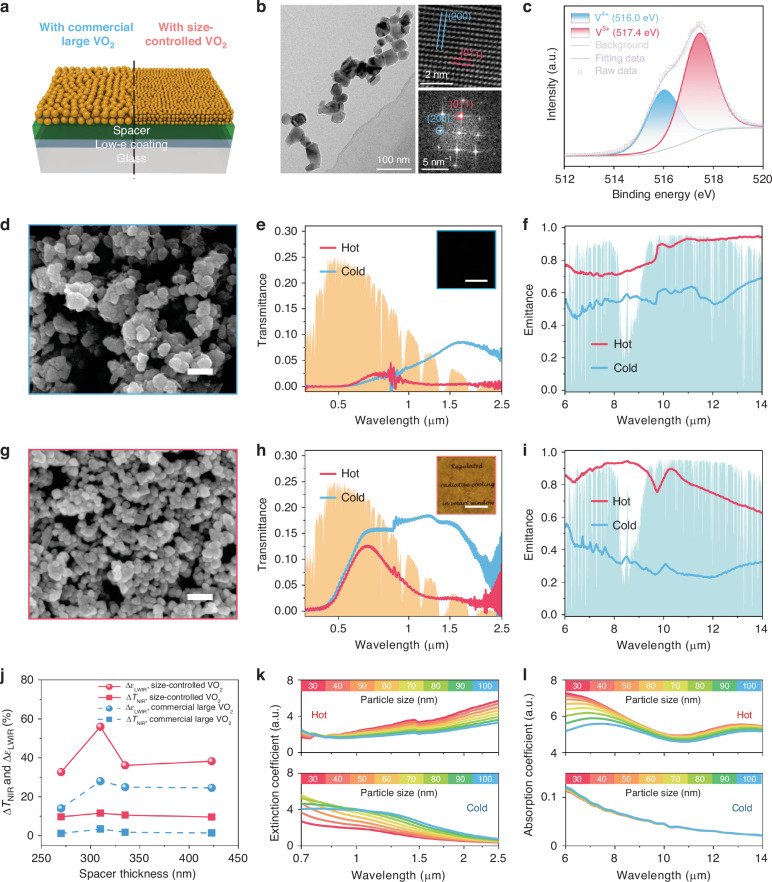
Preparation and characterization of the DPP smart window. **a** The schematic of the smart window with larger commercial VO_2_ NPs (left) and size-controlled NPs (right). **b** TEM image of the size‑controlled VO_2_ NPs, HRTEM image of lattice fringes, and corresponding FFT pattern. **c** V 2*p* core level XPS spectra of size-controlled VO_2_ NPs. **d** SEM image of VO_2_ NPs with a larger particle size. Scale bar: 100 nm. **e**, **f** Transmittance (**e**) and emittance (**f**) spectra of a DPP smart window with a larger VO_2_ particle size. Scale bar: 100 nm. The inset shows the optical image of the DPP smart window. **g** SEM image of VO_2_ NPs with controlled size. Scale bar: 100 nm. **h**, **i** Transmittance (**h**) and emittance (**i**) spectra of a DPP smart window with size-controlled VO_2_ NPs. **j** Δ*T*_NIR_, and Δ*ε*_LWIR_ of the smart window incorporating VO_2_ particles with controlled size, compared with commercial large VO_2_ particles. **k** Extinction coefficient of VO_2_ NPs in hot (top) and cold (bottom) environments, respectively. **l** Absorption coefficient of VO_2_ NPs in hot (top) and cold (bottom) environments, respectively

The ML findings suggest that optimal DPP smart window performance requires VO_2_ particle sizes below 40 nm. We thus fabricated size‑controlled VO_2_ NPs via a solvothermal-annealing route using commercial VO_2_ particles of 70 nm as a benchmark. (Fig. 3a). Transmission electron microscopy (TEM) image reveals a uniform distribution of NPs (Fig. 3b). The corresponding high-resolution transmission electron microscopy (HRTEM) image displays distinct lattice fringes with interplanar spacings of 0.24 nm and 0.32 nm, which can be indexed to the (200) and (011) crystal planes of VO_2_(M), respectively (Fig. 3b). The associated Fast Fourier Transform (FFT) pattern further confirms the presence of these crystal planes (Fig. 3b). X-ray diffraction (XRD) pattern shows sharp, high-intensity diffraction peaks for the size-controlled VO_2_ NPs, corresponding well to the standard JCPDS card #82-0661 for VO_2_(M) (Fig. S11). The X-ray photoelectron spectroscopy (XPS) spectrum of the V 2*p* core level shows two characteristic peaks at binding energies of 516.0 eV and 517.4 eV, corresponding to V^4+^ and V^5+^ oxidation states, respectively (Fig. 3c). The presence of the V^5+^ is primarily attributed to surface oxidation of the NPs during ambient storage. Differential scanning calorimetry (DSC) shows a pronounced, reversible phase transition with clear endothermic and exothermic peaks upon heating and cooling, corresponding to a latent heat of ~37 J/g (Fig. S12). Notably, commercial large VO_2_ NPs give comparable latent heat (~40 J/g, Fig. S13).

These two types of VO_2_ NPs with different spacer thicknesses were used to investigate the modulation capability of the FP smart window. The polyimide (PI) spacer was first formed by spin-coating a PI solution onto cleaned transparent low-e ITO glass (Fig. S14), followed by stepwise thermal curing in a temperature-gradient oven (Fig. S15). The spacer thickness was controlled via spin speed (Fig. S16), with thickness ranging from 200 to 430 nm. PI coatings of these thicknesses on the ITO substrate did not affect the low‑e characteristics of the ITO glass (Fig. S17), confirming their effectiveness as LWIR-transparent spacers. VO_2_ NPs were mixed in ethanol (Fig. S18), followed by spin-coating onto the cured PI spacer to form a DPP smart window. The thickness of the dense VO_2_ coating was controlled by adjusting the spin speed and the number of spin-coating cycles (Fig. S19). The slight deviation from a perfectly close-packed hexagonal configuration could be due to the complex fluid dynamics and capillary forces during ethanol evaporation. By fixing the spacer thickness of ~310 nm, two particle-size FP devices were fabricated. With large commercial VO_2_ NPs (averaged 70 nm, Figs. 3d and S20), the smart window exhibits a low *T*_lum_ with a limited Δ*T*_NIR_ (Fig. 3e). Its *T*_NIR_ is 4.43% at low temperature and 0.98% at high temperature, giving Δ*T*_NIR_ of 3.45%. In the LWIR emittance spectra, *ε*_LWIR_ is 0.58 at low and 0.86 at high temperature, yielding Δ*ε*_LWIR_ of 0.28 (Fig. 3f). In contrast, the DPP device with size-controlled NPs (35 nm, Fig. 3g and S21) gives tailored *T*_lum_ (<0.15) for DPP, and much enhanced Δ*T*_NIR_ of 11.6% and Δ*ε*_LWIR_ of 0.56 with its *T*_NIR_ of 4.7% and *ε*_LWIR_ of 0.83 at high temperature, and *T*_NIR_ of 16.3% and *ε*_LWIR_ of 0.27 at low temperature (Fig. 3h, i). A similar trend was observed across different spacer thicknesses for 270, 335, and 423 nm spacers (Figs. S22–S24). Smaller particle size enhances both Δ*ε*_LWIR_ and Δ*T*_NIR_ (Fig. 3j), and this observation aligns well with the ML prediction (Fig. 2e–g).

The Wave Optics Module in COMSOL Multiphysics software is further simulated to elucidate the mechanism of VO_2_ electromagnetic‑field responses at both NIR and LWIR wavelengths, with particle diameters ranging from 30 to 100 nm in both the metallic VO_2_(R) and insulating VO_2_(M) states. The simulation model is shown in Fig. S25. We extracted the size-dependent extinction efficiency *Q*_ext_, which inversely correlates with transmittance, and the absorption coefficient *Q*_abs_, which is directly correlated to emittance. In the hot state, smaller particles exhibit larger *Q*_ext_ in the NIR spectrum, whereas in the cold state, they exhibit smaller *Q*_ext_. Thus, decreasing particle size improves *T*_NIR_ in the cold state and lowers it in the hot state, resulting in increased Δ*T*_NIR_ (Fig. 3k). Likewise, *Q*_abs_ increases with decreasing particle size in the hot state but shows negligible size dependence at the cold state, yielding a larger Δ*ε*_LWIR_ for smaller NPs (Fig. 3l). The consistency between the optical simulation trends and the ML predictions validates our inverse design framework. It demonstrates that the ML model successfully captured the underlying physics, which guides the structure design and synthesis towards optimal structures.

### Performance evaluation

**Fig. 4:**
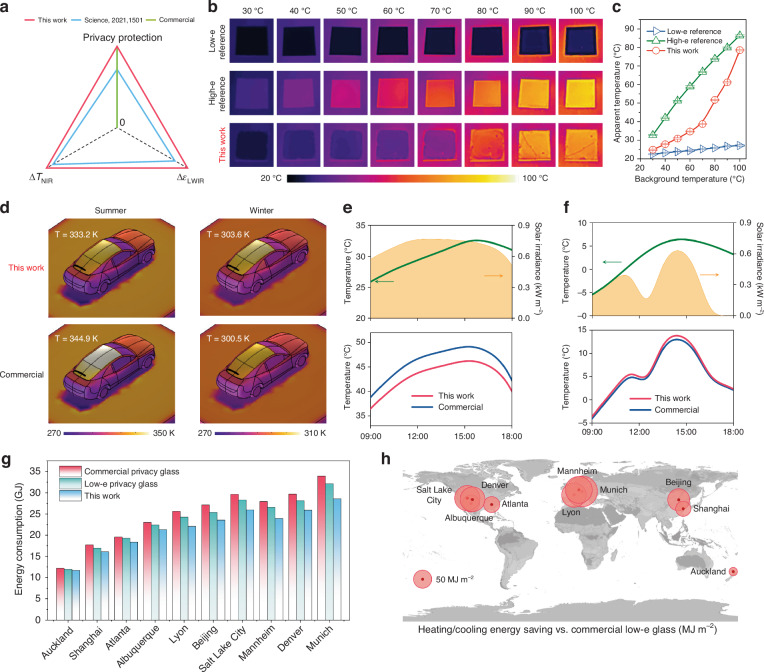
Performance evaluation of the DPP smart window. **a** Comparison of privacy-protection performance, Δ*T*_NIR_, and Δ*ε*_LWIR_ among the present work, commercial glass, and reported results^8^. **b** IR camera image of the low-e reference, high-e reference, and the DPP smart window. **c** Apparent temperature measured over 30 ~ 100 °C. **d** Temperature distribution across the entire car body on a typical summer day (left) and a typical winter day (right), obtained from simulations with the DPP smart window (top) and with commercial glass (bottom). The averaged surface temperatures (*T*) of the used glass are shown in the upper-left corner. **e** Ambient temperature and solar irradiance on a typical summer day (top). The mean interior car temperature for the two glazing configurations (DPP smart window vs commercial privacy glass) (bottom). **f** Ambient temperature and solar irradiance on a typical winter day (top). The mean interior car temperature for the two glazing configurations (DPP smart window vs commercial privacy glass) (bottom). **g** Simulated building energy consumption for commercial privacy glass, low-e privacy glass, and the DPP smart window. **h** Annual heating and cooling energy-saving potential of the DPP smart window compared to low-e privacy glass in ten mid-latitude cities

The DPP smart window based on size‑controlled VO_2_ NPs exhibits an overall optical performance with customized *T*_lum_ < 0.15 and large tunability (Δ*T*_NIR_ = 0.12 and Δ*ε*_LWIR_ = 0.56), outperforming commercial privacy glass and surpassing representative prior reports (Δ*T*_NIR_ = 0.10 and Δ*ε*_LWIR_ = 0.4)^8^ (Fig. 4a, Table S7).

To further evaluate its thermal management capability, IR thermography was conducted on the DPP device and two reference samples, including a low-e metal film (*ε*_LWIR_ ~ 0.1) and a high-e glass (*ε*_LWIR_ ~ 0.9), under a background temperature sweep from 30 to 100 °C (Fig. 4b). At the same background temperature, darker colors correspond to lower *ε*_LWIR_ and brighter colors to higher *ε*_LWIR_. The IR images clearly indicate that the DPP smart window functions as an IR reflector at low temperature, similar to the low-e reference, and transitions into an IR emitter at high temperature analogous to the high-e reference. The corresponding apparent temperatures plotted in Fig. 4c demonstrate a distinct transition of the DPP smart window from a low-e state to a high-e state as the ambient temperature increases.

To evaluate the application of the DPP smart window in electric vehicles, we constructed a three-dimensional car model (Fig. S26), incorporating multi-physics coupling, including solid conduction and surface-to-surface radiative heat transfer. Steady-state simulations were performed to assess the temperature field distribution inside the vehicle, using typical climate conditions for mid-latitude regions during summer and winter noon (Table S8). Commercial glass and the DPP smart window were applied to the rear, roof and back side windows of the vehicle (Fig. 4d) with the surface-averaged temperatures calculated. At summer noon, the DPP smart window exhibits a significantly lower temperature than the commercial glass (333.2 K vs. 344.9 K), indicating better passive cooling potential. Conversely, at winter noon, the DPP smart window demonstrates a higher temperature than the commercial glass (303.8 K vs. 300.5 K), reflecting its potential to offer better warming effect. To further investigate the impact of both types of glass on the interior temperature, we simulated the interior air temperature under meteorological conditions typical of a hot summer day in Beijing, China, specifically accounting for ambient temperature, solar irradiance, and wind speed (Fig. 4e and S27a). With the DPP smart window, the indoor air temperature is up to 3 °C lower than the counterpart, giving an average difference of 2.1 °C. During winter (Fig. 4f and S27b), the DPP smart window results in a maximum increase of 0.9 °C in indoor air temperature, giving an average difference of 0.3 °C. This passive thermal regulation reduces HVAC demand and may extend the driving range of electric vehicles.

To evaluate the energy-saving potential of the fabricated DPP smart window in building applications (Fig. S28), whole-building energy consumption simulations were performed using the EnergyPlus software (Table S9)^8,32^. The simulations assessed the annual heating and cooling energy consumption of buildings in 10 cities located in mid-latitude regions where seasonal adaptation is needed (Table S10). Three types of glasses were considered (Table S11), including commercial privacy glass, low-e privacy glass, and the DPP smart window while keeping *T*_lum_ at ~0.1. DPP smart window outperforms the other two counterparts (Fig. 4g). Taking Beijing as an example, the total energy consumption of the building with commercial privacy glass, low-e privacy glass, and DPP smart window is 27.2, 25.4, and 23.6 GJ, respectively. To better understand the energy-saving potential of the DPP smart window, its performance was compared to that of commercial low-e privacy glass across 10 cities (Fig. 4h). The DPP smart window delivered consistently better energy savings across all cities, with the highest energy savings of 145.8 MJ m^−2^ and the largest improvement of 10.9% in Munich (Germany). Collectively, these results yield an average energy saving of 74.2 MJ m^−2^ across the mid-latitude regions, representing 7% of total energy consumption, attributable to the DPP window’s passive, temperature-dependent dynamic thermal regulation. Furthermore, comparisons with the state-of-the-art window^8^ confirm the superior energy efficiency of the DPP smart window (Fig. S29).

## Discussion

In summary, we present a physics‑guided neural‑network framework for the inverse design of function-oriented thermochromic smart windows, specifically aiming for directional privacy and energy savings. The model identifies that relatively small VO_2_ NPs are needed for both optimized Δ*ε*_LWIR_ and Δ*T*_NIR_, while spacer thickness shows a negligible effect on Δ*T*_NIR_ but needs to be tuned for the Δ*ε*_LWIR_ due to the enhanced FP resonance. Aided by these predictions, we synthesized size‑controlled VO_2_ NPs and incorporated them into DPP smart windows, achieving a high Δ*T*_NIR_ of 0.12 and Δ*ε*_LWIR_ of 0.56, surpassing commercial glass and the cutting-edge results. Superior performance was demonstrated in both electric vehicles and building envelopes. Compared with commercial counterparts, the DPP smart windows reduce electric-vehicle cabin peak air temperature by up to 3 °C in summer and increase 0.9 °C in winter, while building-energy simulations indicates 7% of energy savings in mid-latitude climate buildings. This physics-guided inverse-design strategy establishes a generalizable route to energy-efficient smart windows with customized optical responses required for real-world deployment.

## Materials and methods

### Materials

Large VO_2_ NPs were purchased from Hangzhou Jikang New Material CO., LTD. Vanadyl (IV) acetylacetonate (VO(acac)_2_, 99%) was purchased from RHAWN and used as the vanadium source without further purification. Benzyl alcohol (C_7_H_8_O, 99%) and ethanol (analytical grade) were obtained from Sinopharm Chemical Reagent Co., Ltd. N,N-Dimethylformamide (DMF, ≥99.8%, analytical grade) was used as the solvent for preparing the polyamic acid (PAA) precursor solution. The PAA powder was supplied by Fuxin Hongyang Optoelectronic Materials Co., Ltd. and served as the precursor for the fabrication of polyimide (PI) spacer films. Commercial ITO low-e glass was purchased from Foshan Shi Yuan Jing Mei Glass Co., Ltd. and used as a substrate. High-purity oxygen gas (O_2_, 99.999%) was used during the annealing process to control the oxidation environment. Deionized water was produced in the laboratory and used for all cleaning and rinsing procedures. Unless otherwise stated, all reagents were used as received without any additional purification.

### Synthesis of VO_2_ NPs

VO_2_ NPs were synthesized using a solvothermal–annealing route. In a typical procedure, 13.258 g of VO(acac)_2_ was completely dissolved in 150 mL of benzyl alcohol under continuous magnetic stirring to form a homogeneous solution. The solution was transferred into a 250 mL Teflon-lined stainless-steel autoclave, sealed, and heated at 200 °C for 8 h. After naturally cooling to room temperature, the resulting product was collected by centrifugation. The precipitate was repeatedly washed with ethanol to remove any unreacted organics and then dried under vacuum at 70 °C. The dried solid precursor was subjected to annealing in a horizontal tube furnace under a continuous oxygen flow. The sample was heated to 370 °C and maintained for 1 h, followed by natural cooling to ambient temperature. This treatment converted the precursor into highly crystalline VO_2_(R) NPs with thermochromic phase transition properties.

### Preparation of the spacer and VO_2_ coating

Polyamic acid (PAA) powder (1.8 g) was dissolved in 30 mL of N,N-dimethylformamide (DMF) to obtain a homogeneous polymer solution. The mixture was magnetically stirred at 35 °C for 5 h in an oil bath to ensure complete dissolution. Subsequently, the resulting viscous solution was degassed under vacuum for 30 min to remove any trapped air bubbles. Commercial ITO low-e glass substrates were cleaned before coating. Each substrate was ultrasonically treated for 10 min in acetone, followed by ethanol and deionized water, and finally dried under a stream of high-purity nitrogen gas. The PAA precursor solution was then deposited onto the cleaned ITO substrates by spin-coating (3500 rpm) to form a uniform wet film. The coated substrates were subjected to a stepwise thermal curing process in air to induce imidization of the PAA. The temperature was gradually increased from 30 °C to 180 °C, holding at each temperature for 30 min. After completion of the thermal cycle, the samples were allowed to cool naturally to room temperature, yielding smooth and compact PI spacer films with good adhesion to the ITO substrate. Finally, 1 g of VO_2_ NPs was uniformly dispersed in 10 mL of ethanol. After ultrasonication and thorough mixing, a stable suspension was obtained. This dispersion was then spin-coated onto the PI surface, leading to the formation of a VO_2_ layer. The spin-coating process was optimized to achieve uniform coverage and the desired film thickness.

### Characterization

The cross-sectional morphology of the samples was examined using a scanning electron microscope (FEI Nova Nano SEM 450). The particle size distribution of the VO_2_(R) NPs was statistically analyzed using Nano Measure. The electrical performance of the films was evaluated by measuring their sheet resistance with a four-probe resistivity tester (RTS-3) at room temperature. Optical characterization was carried out using a UV-VIS-NIR spectrophotometer (Shimadzu UV-3600 Plus) equipped with an integrating sphere attachment (ISR-603) to record transmittance spectra in the wavelength range of 0.3 ~ 2.5 μm. The MIR emittance of the samples was determined using a Fourier-transform infrared spectrometer (FTIR, Thermo Fisher SCIENTIFIC, Nicolet-is50, America) equipped with a gold integrating sphere (MCT/A Detector, PIKE, America) to ensure accurate hemispherical emissivity measurements. HRTEM (Talos F200s, USA) was used to characterize the microstructure of the NPs. Elemental composition and valence state of the NPs were determined by XPS (K-Alpha, Thermo Fisher Scientific, USA). The crystal phase composition was determined by X-ray diffraction (XRD, Model D/Max 2550 V, Rigaku, Japan). The thermal phase transition behavior was analyzed by differential scanning calorimetry (DSC, Q2000, TA Instruments, America). The thickness of the coating was measured with a stylus profilometer (DektakXT, Bruker, America). The infrared images were captured by the infrared camera (TiX520, Fluke, America) to assess the thermal properties of the samples.

### Calculating from the spectrum

According to Kirchhoff’s law, the sum of reflectance, transmittance, and absorption equals 1. We utilized the following equations to calculate the integral solar transmittance (*T*_sol_), luminous transmittance (*T*_lum_), near-infrared transmittance (*T*_NIR_) and LWIR emissivity (*ε*_LWIR_), as expressed by Equations 1–4, respectively^33,34^.

1 $${T}_{\mathrm{sol}}=\frac{{\int }_{0.3}^{2.5}d\lambda \cdot t\left(\lambda \right)\cdot {I}_{{AM}.1.5}\left(\lambda \right)}{{\int }_{0.3}^{2.5}d\lambda \cdot {I}_{{AM}.1.5}\left(\lambda \right)}$$

2 $${T}_{\mathrm{lum}}=\frac{{\int }_{0.38}^{0.78}d\lambda \cdot t\left(\lambda \right)\cdot {\varphi }_{{lum}}\left(\lambda \right)}{{\int }_{0.38}^{0.78}d\lambda \cdot {\varphi }_{{lum}}\left(\lambda \right)}$$

3 $${T}_{\mathrm{NIR}}=\frac{{\int }_{0.78}^{2.5}d\lambda \cdot t\left(\lambda \right)\cdot {I}_{{AM}.1.5}\left(\lambda \right)}{{\int }_{0.78}^{2.5}d\lambda \cdot {I}_{{AM}.1.5}\left(\lambda \right)}$$

4 $${\varepsilon }_{\mathrm{LWIR}}=\frac{{\int }_{8}^{13}d\lambda \cdot (1-r\left(\lambda \right))\cdot {I}_{{BB}}\left(T,\lambda \right)}{{\int }_{8}^{13}d\lambda \cdot {I}_{{BB}}\left(T,\lambda \right)}$$

where *I*_AM1.5_(λ) represents the solar illumination of the standard AM 1.5 spectrum and *I*_BB_ (T, λ) denotes the spectral radiance of a blackbody at temperature *T*. *φ*_lum_(λ) is the standard luminous efficiency function of photopic vision for the wavelength of 380 ~ 780 nm. The variables *t*(*λ*) and *r*(*λ*) correspond to the measured transmittance and reflectance within the relevant wavelength range, respectively.

### Optical and thermal simulation

Numerical simulations of the optical response were performed using the finite element method (FEM) implemented in COMSOL Multiphysics. The scattering and absorption efficiencies of the VO_2_ NPs were systematically analyzed to elucidate their interaction with electromagnetic radiation. The computational model consisted of a representative unit cell enclosed by perfectly matched layers (PMLs) along the propagation direction to suppress non-physical reflections. Periodic boundary conditions were imposed along the lateral directions to mimic an infinite periodic arrangement, thereby capturing the collective optical characteristics of the nanostructure. Two port boundaries were defined at the top and bottom of the simulation domain to introduce and detect the propagating electromagnetic waves. The excitation source was modeled as a linearly polarized plane wave. To systematically evaluate the size-dependent optical properties, a parametric sweep was performed for the VO_2_ NP diameters ranging from 30 nm to 100 nm. Concurrently, the simulation wavelengths were swept across two critical regimes: the near-infrared (0.7–2.5 μm) and the long-wave infrared (6–14 μm). All simulations were carried out under steady-state frequency-domain conditions. The exact geometric configuration and boundary setup are illustrated in Fig. S25. The resulting scattering and absorption efficiencies were quantitatively analyzed to interpret and validate the experimental observations. Steady-state temperature simulations were conducted using the Heat Transfer Module in COMSOL Multiphysics, with the Surface-to-Surface Radiation Physics interface employed to model the radiative heat transfer between surfaces. The typical meteorological year (TMY) weather data for Beijing, China (Latitude: 39.8° N, Longitude: 116.5° E) was extracted from EnergyPlus. January 1st and July 21st were selected for the winter and summer conditions, respectively. The geometric model was designed to represent an electric vehicle, with an external radiation source applied to the top surface, simulating realistic environmental conditions. The model accounted for both the emission and absorption properties of the surface, enabling accurate predictions of the temperature distribution across the vehicle’s exterior. Material properties were adjusted based on real-world measurements, ensuring that the simulations reflected actual conditions.

### Energy saving simulation

The building’s energy consumption was assessed using EnergyPlus, a widely recognized whole-building energy simulation software. The building model details are provided in Table S9, with insulation materials meeting the requirements specified in ASHRAE Standard 90.1. The building has a total floor area of 48 m^2^, including a window area of 24 m^2^ with a window-to-wall ratio of 31.75%. The net conditioned area is also 48 m^2^. The optical properties for normal glass, privacy glass, low-e glass, and the DPP smart window used in the simulations are listed in Table S11. It is important to note that the thermochromic behavior of the smart window was simulated using the built-in thermochromic function in EnergyPlus. The interior temperature setpoints for the simulations were 20 °C in winter and 24 °C in summer, in line with previous reports. To minimize calculation deviations, a time step of 10 min was used for the EnergyPlus simulation. Simulations were conducted for ten cities located in mid-latitude regions to predict potential heating energy savings (Table S10), including Auckland (Climate Zone 3 A), Shanghai (Climate Zone 3 A), Atlanta (Climate Zone 3 A), Albuquerque (Climate Zone 4B), Lyon (Climate Zone 4 A), Beijing (Climate Zone 4 A), Salt Lake City (Climate Zone 4B), Mannheim (Climate Zone 5 A), Denver (Climate Zone 5B), and Munich (Climate Zone 5 A). The analysis primarily focused on the energy savings per unit area, which were calculated based on the window glass area.

### ML process

While probabilistic models such as Gaussian Process Regression are traditionally favored in low-data regimes (*N* = 99), a neural network architecture was selected for this framework due to its compatibility with physics-guided multi-objective optimization. First, the neural network supports multi-task learning, utilizing shared hidden layers to capture the intrinsic physical correlations between the dual-band optical responses (Δ*T*_NIR_ and Δ*ε*_LWIR_). Second, the neural network structure allows for the integration of non-linear physical regularizations (e.g., thermodynamic non-negativity and energy conservation boundaries) directly into the loss function topology. Finally, once trained, the neural network provides *O*(1) inference speed, enabling the evaluation of thousands of structural candidates required for inverse design.

A physics-guided artificial neural network was constructed using the TensorFlow framework. The network architecture comprised an input layer receiving two parameters (VO_2_ particle size and spacer thickness), four hidden layers with 64, 128, 64, and 32 neurons, respectively, and an output layer predicting Δ*T*_NIR_ and Δ*ε*_LWIR_. All hidden layers utilized the Swish activation function, with batch normalization and a dropout rate of 0.1 applied to enhance generalization.

The training dataset was initiated from 99 systematically sampled structural configurations of the FP multilayer. FDTD simulations were performed for each configuration to obtain the optical properties under both semiconductor and metallic phases of VO_2_. The simulated structure comprised a bottom ITO glass substrate (ITO thickness: 100 nm), a polymeric spacer layer (thickness: 1.1 ~ 3.1 μm), and a top intrinsic VO_2_ thin film (particle size: 30 ~ 110 nm, thickness: 79 ~ 127 nm). To explicitly prevent any data leakage and ensure a robust evaluation, we implemented a strict “split-then-augment” protocol. The raw dataset was first rigorously partitioned into independent training, validation, and testing sets. Crucially, the physics-informed data augmentation was applied exclusively to the training set, ensuring that the testing partition remained completely unseen during the entire model optimization process.

Importantly, this physics-informed noise injection also serves to bridge the simulation-to-experiment gap. While FDTD simulations assume perfectly spherical VO_2_ NPs, realistic chemical synthesis often yields particles with slight morphological irregularities. By applying stochastic perturbations to the effective diameter during training, we force the physics-guided neural network to learn a mapping that is tolerant to such fabrication-induced shape variations.

Prior to network training, all input geometric parameters and output optical targets underwent Z-score standardization (${x}^{{\prime} }=(x-\mu )/\sigma$). The scaling parameters ($\mu$ and $\sigma$) were fitted on the training subset to prevent data leakage. This normalization protocol maps features with disparate physical units and numerical scales (e.g., nanometers vs. micrometers) into a uniform dimensionless space with zero mean and unit variance, thereby preventing optimization bias and supporting efficient convergence during gradient descent. The model was trained using the Adam optimizer with an initial learning rate of 10^−3^ and employed an early stopping callback (patience = 150) on the validation loss to prevent overfitting.

### Physics-constrained loss formulation

To ensure the model acts as a physics-guided neural network rather than a conventional data-driven black box, we incorporated physical constraints into the training formulation. While data augmentation expands the training distribution, it does not prevent a standard neural network from predicting physically invalid behaviors in unseen geometrical configurations. Therefore, we structured our total loss function (${L}_{{\rm{total}}}$) to encompass both empirical data fitting and physical regularization:

5 $${L}_{{total}}=\frac{1}{N}\displaystyle \mathop{\sum }\limits_{i=1}^{N}{({y}_{i}-{\hat{y}}_{i})}^{2}+{\lambda }_{{phy}}{{L}}_{{phy}}({\hat{y}}_{i})+{\lambda }_{{smooth}}{{L}}_{{smooth}}({\hat{y}}_{i},{{\boldsymbol{x}}}_{i})$$

The first term represents the standard mean squared error. The second term, ${{L}}_{{phy}}$, enforces thermodynamic directionality and energy conservation boundaries. Specifically, the near-infrared modulation ability (Δ*T*_NIR)_ and emissivity variation (Δ*ε*_LWIR_) induced by the VO_2_ phase transition must be non-negative and cannot exceed 100%. We implemented this via a Rectified Linear Unit penalty mechanism:

6 $${{L}}_{{phy}}={\rm{ReLU}}(-\hat{y})+{\rm{ReLU}}(\hat{y}-1.0)$$

This constraint ensures the predicted optical responses reside within the valid physical interval [0, 1].

Furthermore, the third term, ${{L}}_{{smooth}}$, enforces physical continuity. To prevent the model from capturing numerical noise or exhibiting abrupt oscillations in the predicted spectra, we applied a smoothness regularization by penalizing the squared L2-norm of the output gradients with respect to the input geometric features (${{\boldsymbol{x}}}_{i}$):

7 $${{L}}_{{smooth}}=||{\nabla }_{x}{\hat{y}}_{i}|{|}^{2}$$

The hyperparameters ${\lambda }_{{phy}}$ and ${\lambda }_{{smooth}}$ were utilized to balance the trade-off between data fidelity and physical compliance. Through this physics-constrained optimization landscape, the physics-guided neural network is bounded by fundamental optical laws, ensuring reliable and physically consistent inverse design evaluations.

Permutation feature importance analysis was performed to quantify the relative influence of VO_2_ particle size and spacer thickness on each optical performance metric. The analysis focused on parameter ranges of 30 ~ 100 nm for particle diameter and 1.1 ~ 2.1 μm for spacer thickness, representing regions of practical relevance and significant performance variation observed in forward simulations.

For the inverse design, a normalized scoring function was defined:

8 $$S=0.5\times \left(\frac{\Delta {\varepsilon }_{LWIR}}{\Delta {\varepsilon }_{LWIR\_\max }}+\frac{\Delta {T}_{NIR}}{\Delta {T}_{NIR\_\max }}\right)$$

where ∆*ε*_LWIR_max_ and ∆*T*_NIR_max_ represent the maximum achievable values for each performance metric. A comprehensive search of 5000 physically realizable candidate configurations within the parameter space was conducted to identify designs maximizing S. Pareto front analysis was subsequently performed to elucidate the fundamental trade-off relationship between ∆*ε*_LWIR_ and ∆*T*_NIR_ performance objectives.

## Supplementary information

Supplementary Information for Machine Learning-Assisted Highly Efficient Thermal Management in Function-Oriented Thermochromic Smart Windows

## Data Availability

The raw experimental datasets analyzed during the current study, along with the complete source code for the physics-guided neural network framework, are available. The code repository includes comprehensive scripts for data preprocessing, physics-informed data augmentation, and the physics-constrained model training pipeline. The code and data can be accessed via https://github.com/StarGazerLua/ML-TC-Window.git.
