# Supplementary material for: Machine learning-assisted highly efficient thermal management in function-oriented thermochromic smart windows
Source: Light Sci Appl. 2026 Jun 22;15:277. doi: 10.1038/s41377-026-02369-4 (PMC13287441; doi:10.1038/s41377-026-02369-4)
Supplement: Supplementary file 1 — Supplementary Information for Machine Learning-Assisted Highly Efficient Thermal Management in Function-Oriented Thermochromic Smart Windows [file 41377_2026_2369_MOESM1_ESM.docx]

**Supplementary Information for**

**Machine Learning-Assisted Highly Efficient**

**Thermal Management in Function-Oriented Thermochromic Smart Windows**

Zhengui Zhou^1^†, Changyuan Chen^1^†, Bin Li^1^†, Rong Liu^1^, Shouqin Tian^2,*^, Bin Hu^3,4,*^ & Yi Long^1,*^

^1^Department of Electronic Engineering, The Chinese University of Hong Kong, Shatin, New Territories, Hong Kong SAR 999077, China.

^2^State Key Laboratory of Advanced Glass Materials, Wuhan University of Technology, Wuhan 430070, China.

^3^Wuhan National Laboratory for Optoelectronics, School of Optical and Electronic Information, Huazhong University of Science and Technology 430074, Wuhan, China.

^4^Shenzhen Huazhong University of Science and Technology Research Institute, Shenzhen 518057, China.

^*^Correspondence author:

[yilong@cuhk.edu.hk](mailto:yilong@cuhk.edu.hk); [bin.hu@hust.edu.cn](mailto:bin.hu@hust.edu.cn); [tiansq@whut.edu.cn](mailto:tiansq@whut.edu.cn)

†These authors contributed equally to this work

**Supplementary Note 1: Discussion on the Interplay of VO_2_ Particle Size, Layer Number, and Effective Thickness**

In our ML inverse design, the VO_2_ NPs size and the number of particle layers are deliberately coupled rather than treated as independent variables. This setup is driven by the practical requirements of directional privacy protection. To satisfy this preset optical requirement, the total effective thickness of the VO_2_ active layer is bounded. Treating the particle size and layer number as unconstrained parameters would allow the optimization algorithm to generate physically invalid solutions (such as excessively thick or thin layers) that fail the *T*_lum_ criteria. Therefore, coupling these two parameters ensures that the ML optimization remains within valid experimental and application boundaries. Additionally, we note that variations in layer number and particle size alter the packing density and electromagnetic interactions. Our FDTD simulations inherently incorporate these complex multiple scattering events and electromagnetic couplings across all constrained configurations, ensuring that the optimized optical variations could provide a reasonable guideline for practical usage limits.

**Supplementary Note 2: Computational Efficiency Analysis**

To quantify the computational efficiency of the physics-guided neural network surrogate model, we benchmarked the time complexity of our approach against traditional FDTD-based inverse design. As summarized in Supplementary Table 5, relying on 3D FDTD simulations for a full genetic algorithm optimization (assuming 5,000 evaluations) requires approximately 122 days of continuous computation on a standard workstation. By substituting the rigorous FDTD solver with the trained physics-guided neural network, the inference time per structural candidate is reduced from ~35 minutes to milliseconds. This yields a computational acceleration of over 5 orders of magnitude (approx. 10^5^ times faster), transforming the inverse design into a highly efficient process while maintaining physical accuracy.


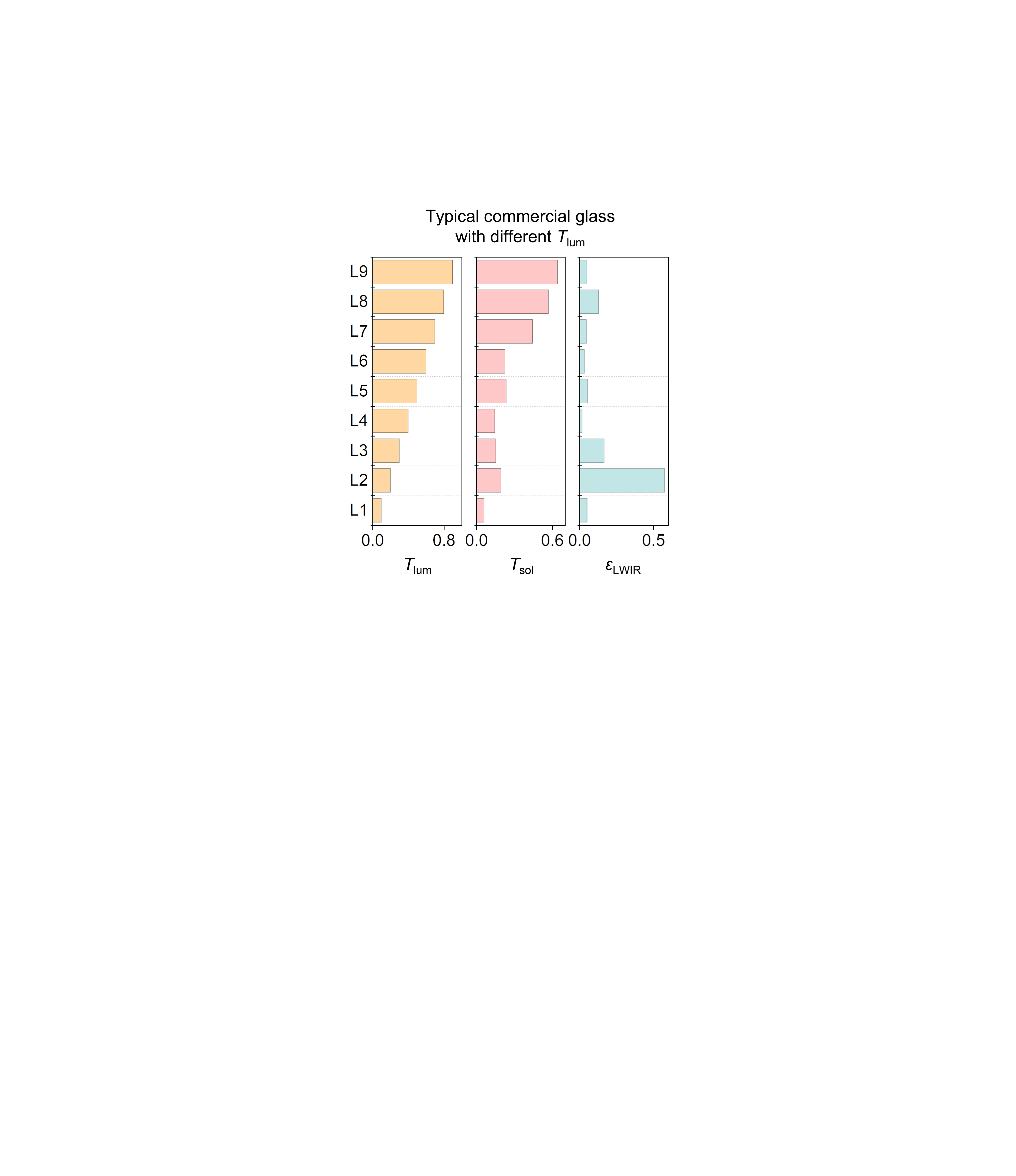


Figure S. *T*_lum_, *T*_sol_ and *ε*_LWIR_ of nine typical commercial glazing products. *T*_lum_ spans 0.1~0.9 across these products, reflecting application-specific requirements.


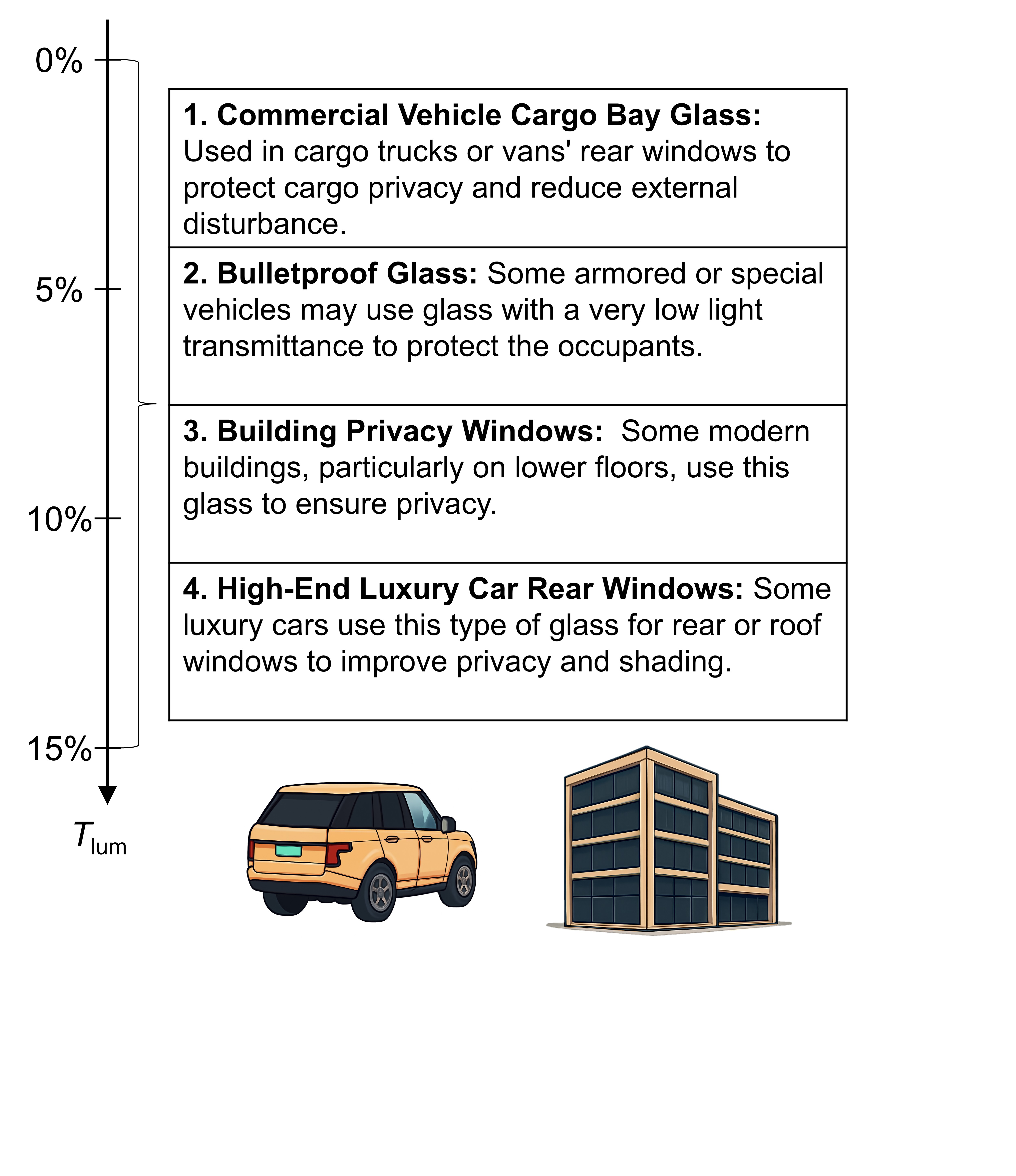


Figure S. Overview of applications for low *T*_lum_ (<0.15) glazing.


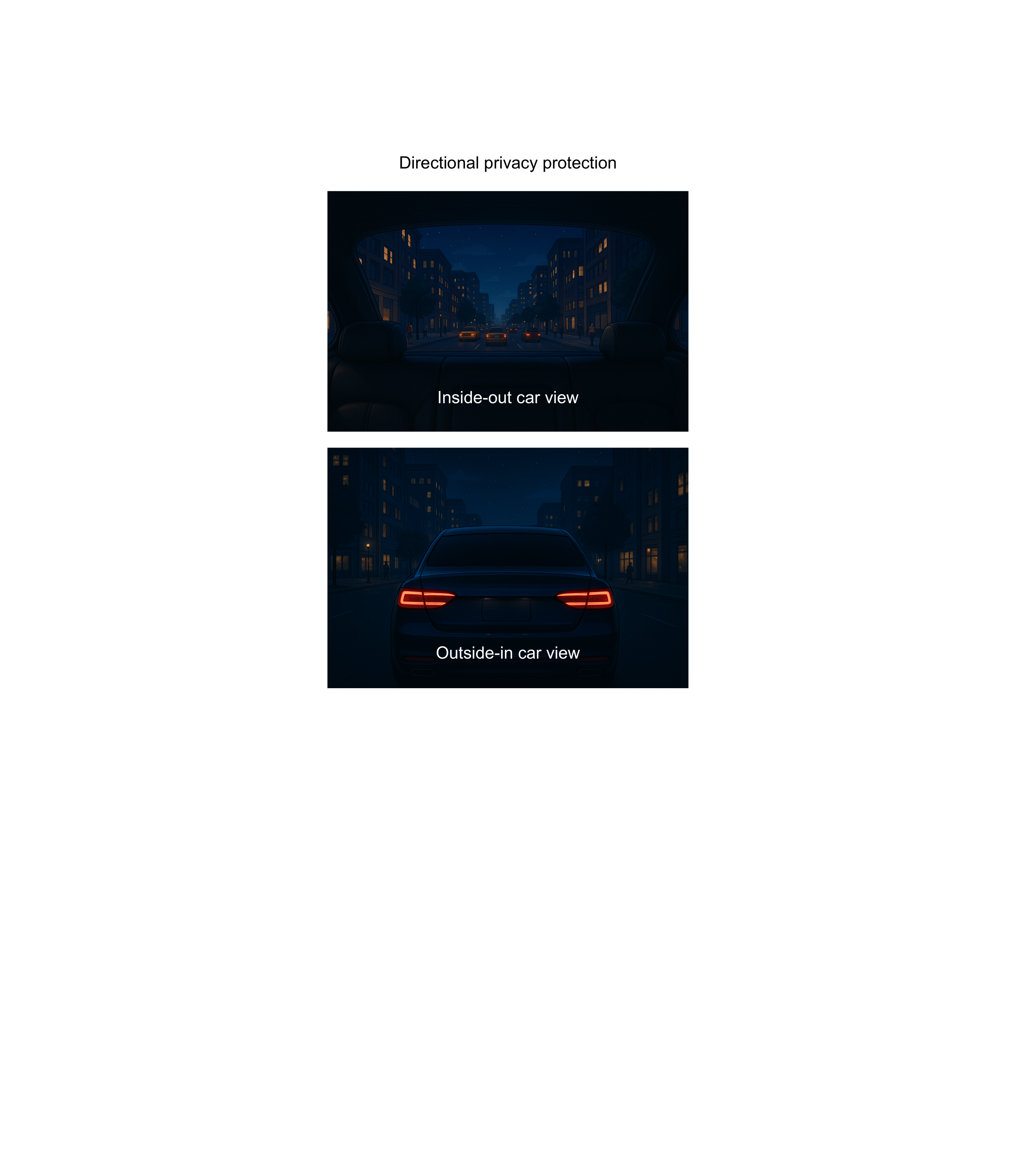


Figure S. Schematic of directional privacy protection at night.


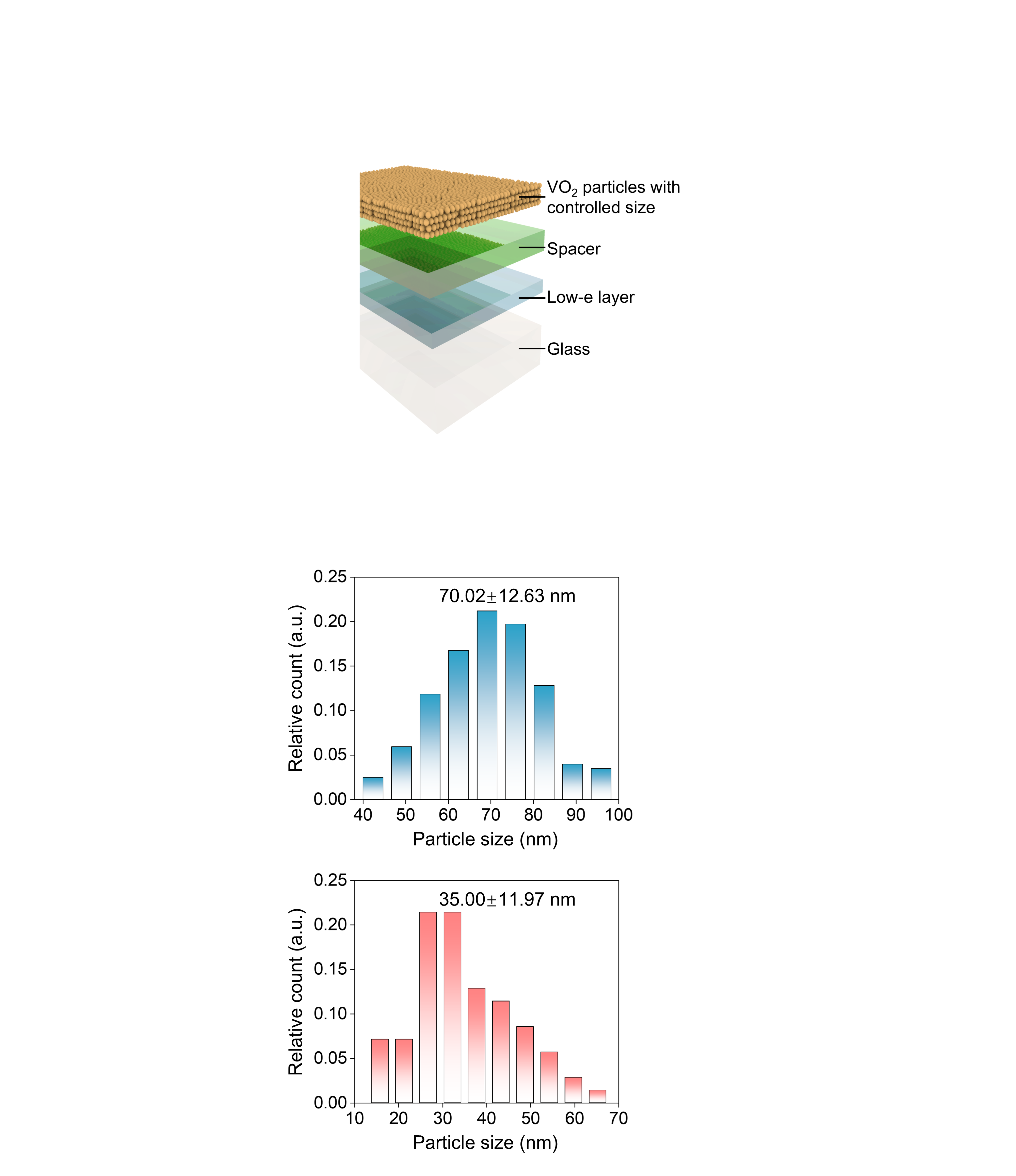


Figure S. Schematic of the multilayer structure of the DPP smart window.


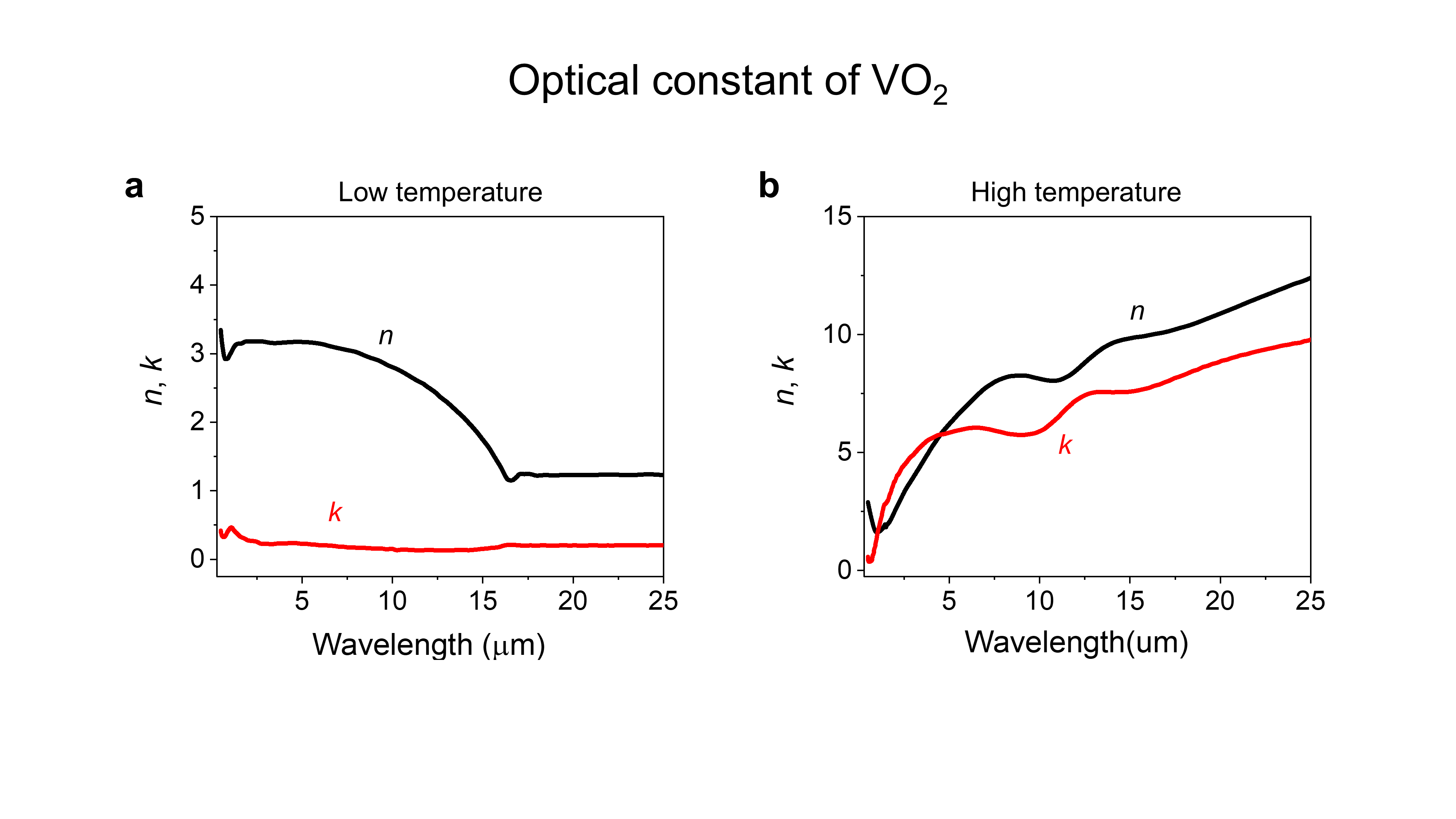


Figure S. Optical constants of VO_2_ at low (a) and high (b) temperatures used in optical simulations^1^.


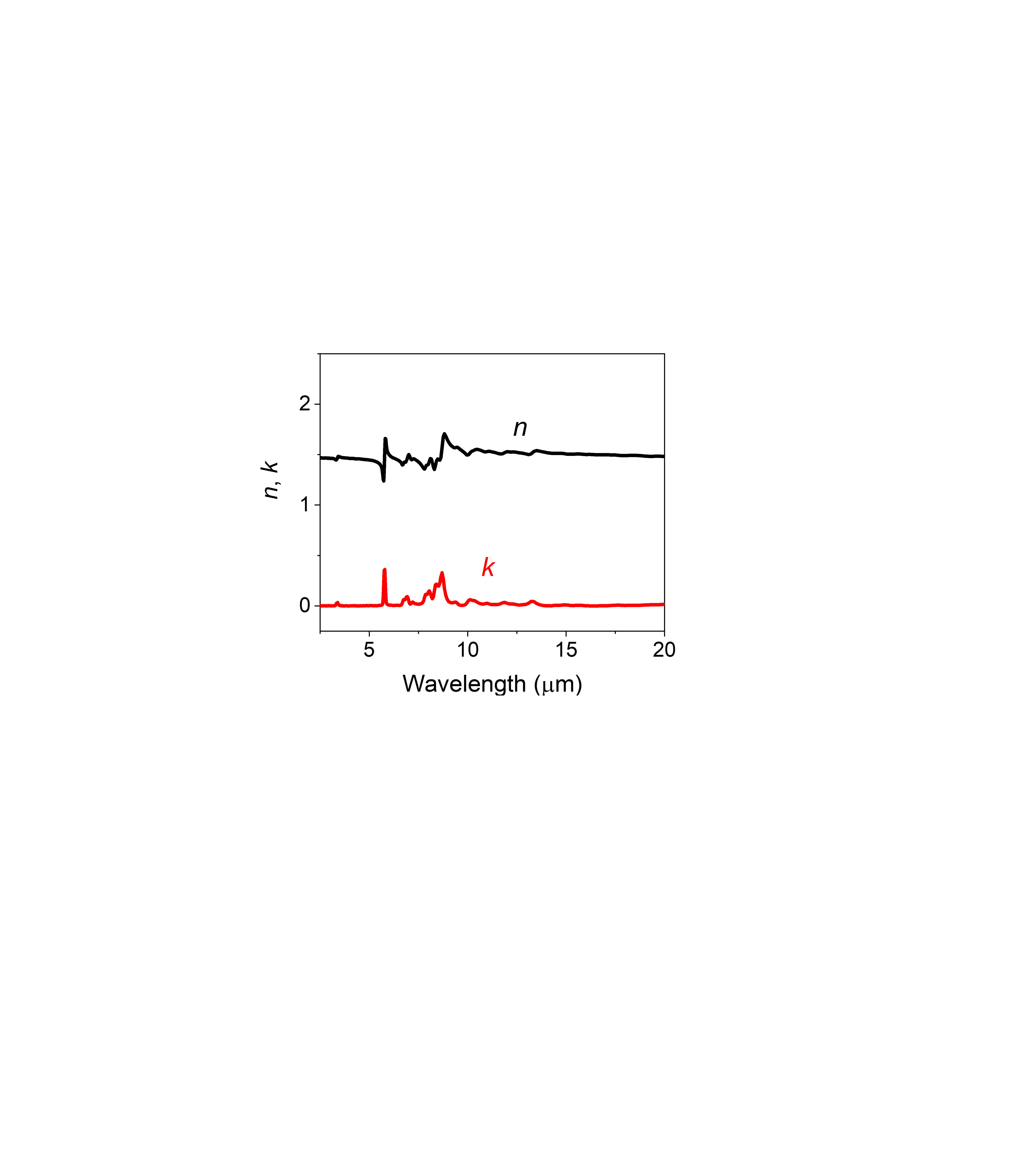


Figure S. Optical constants of polymeric spacer used in optical simulations^1^.


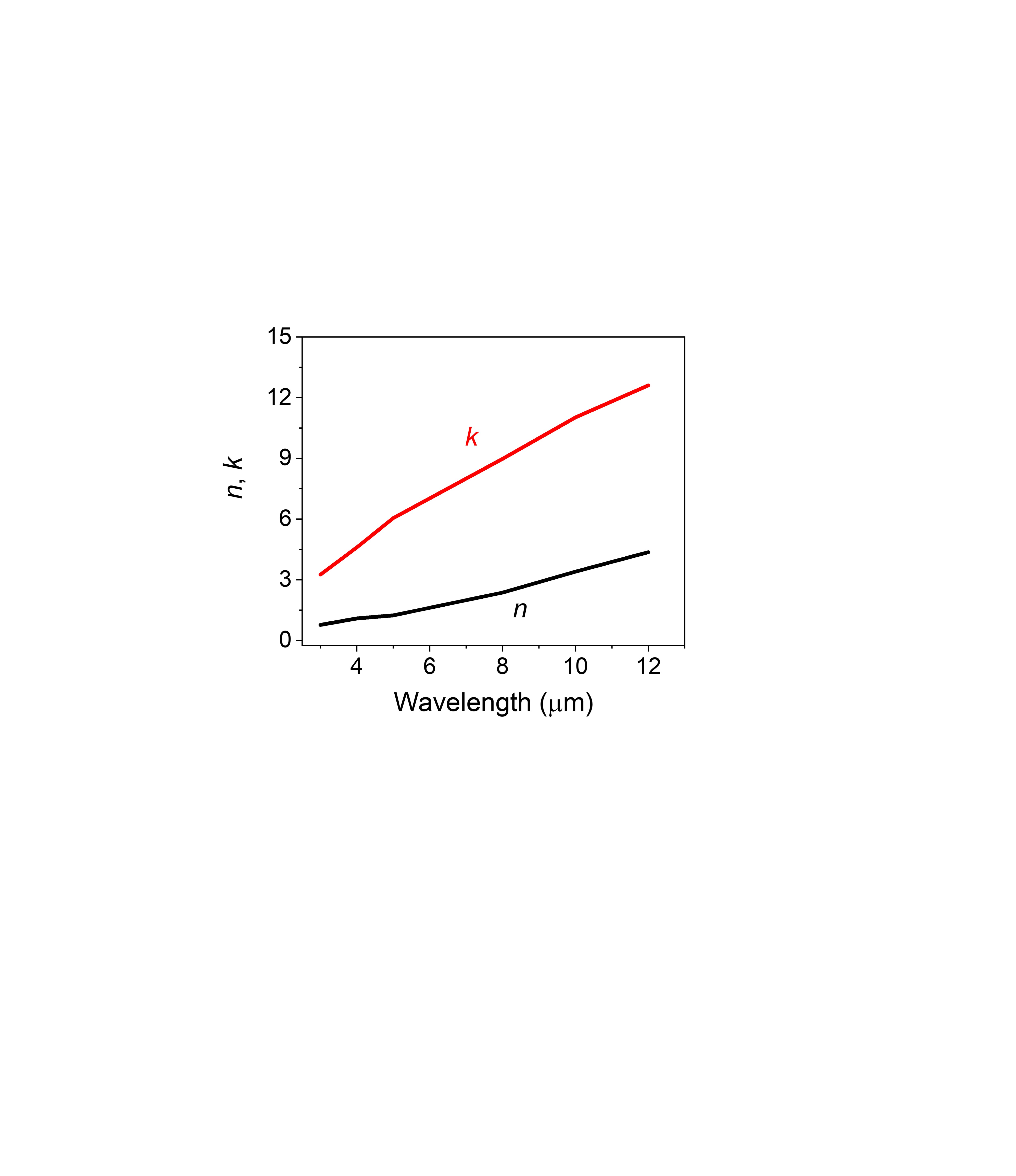


Figure S. Optical constants of ITO layer used in optical simulation^1^.


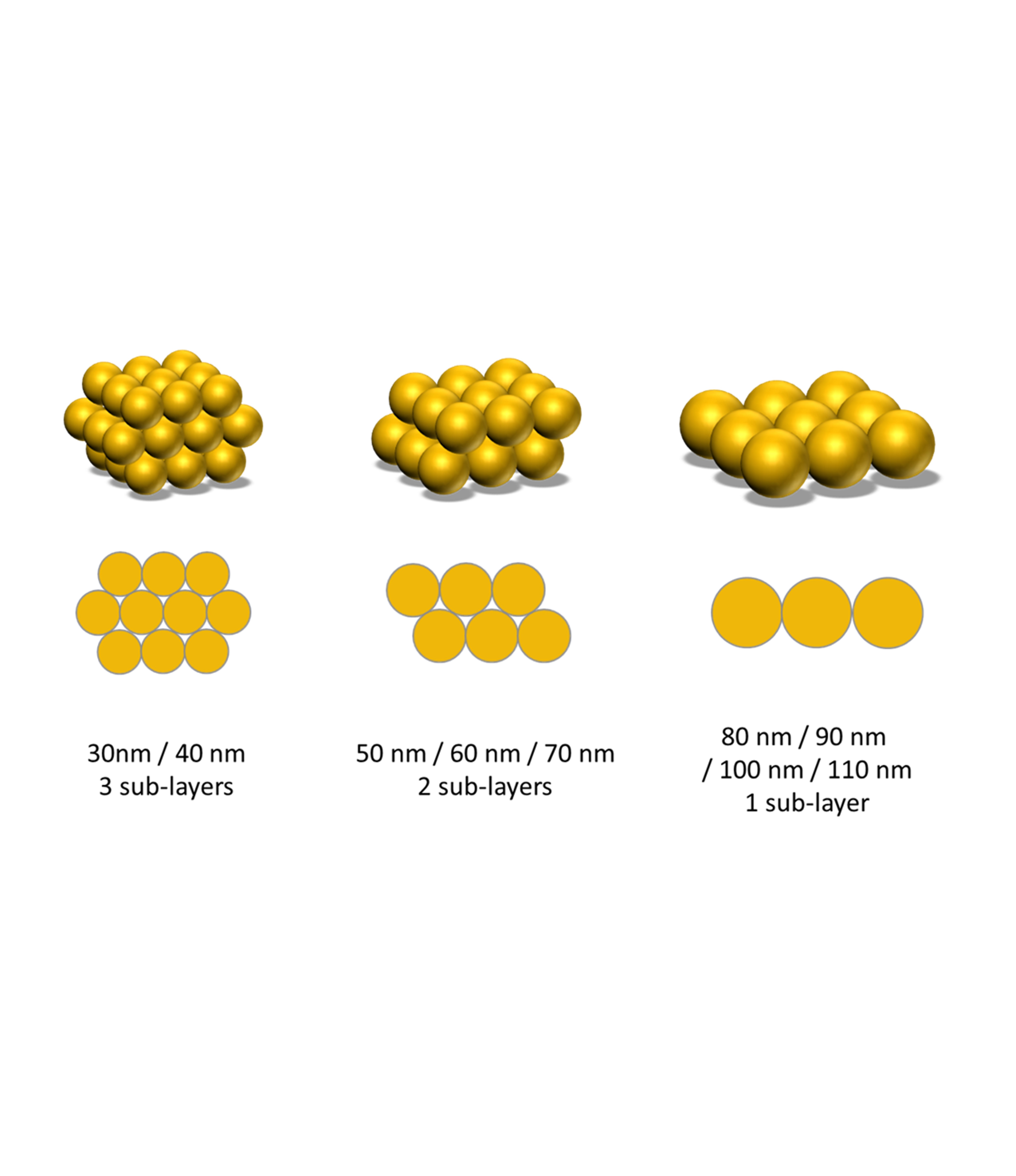


Figure S. Schematic illustration of the hexagonal close-packed VO_2_ nanoparticle configuration.


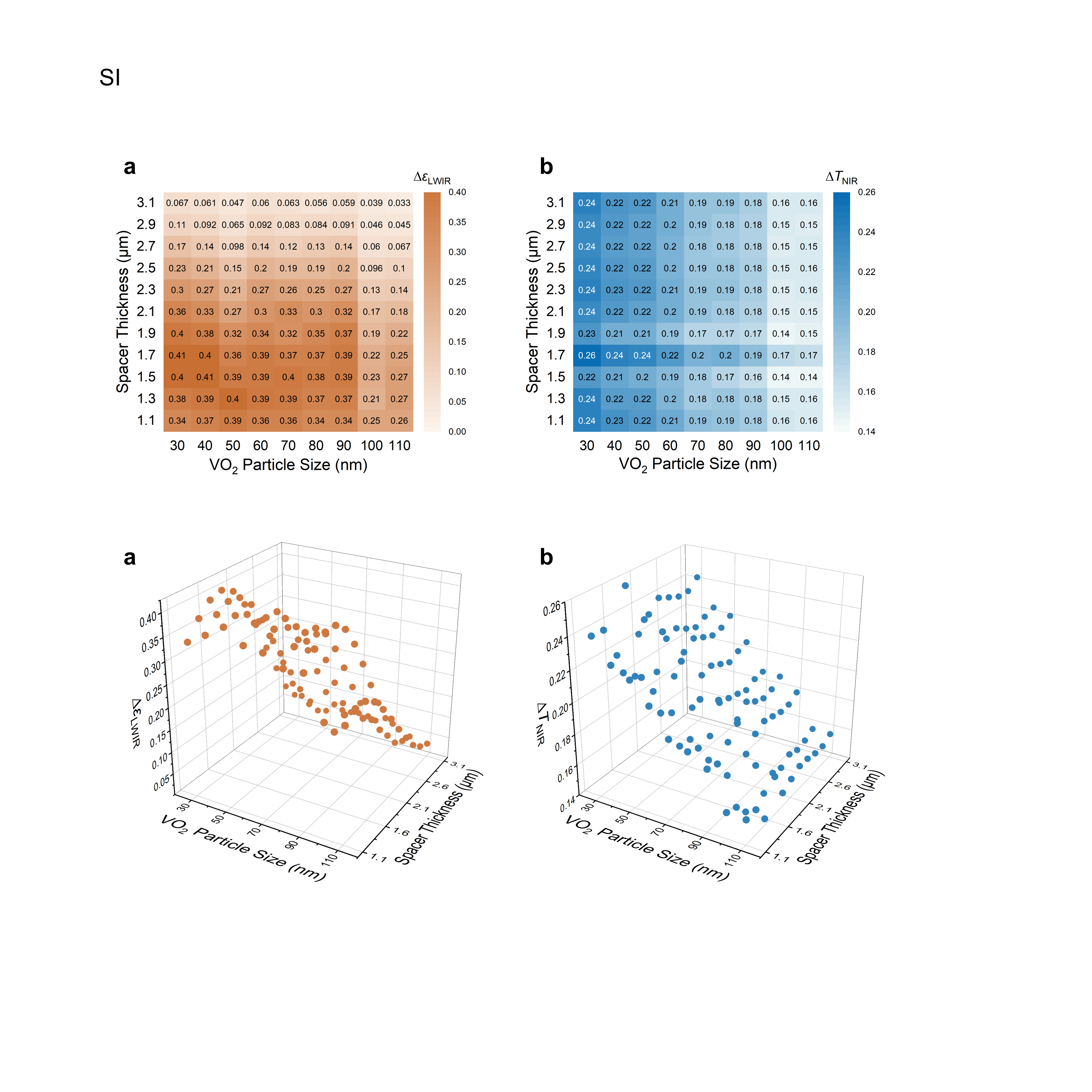


Figure S. Raw FDTD-simulated data for the thermochromic performance metrics, displayed as 2D heatmaps. a, Heatmap of Δ*ε*_LWIR_ as a function of VO_2_ nanoparticle diameter and spacer thickness. b, Heatmap of Δ*T*_NIR_ across the same parameter space.


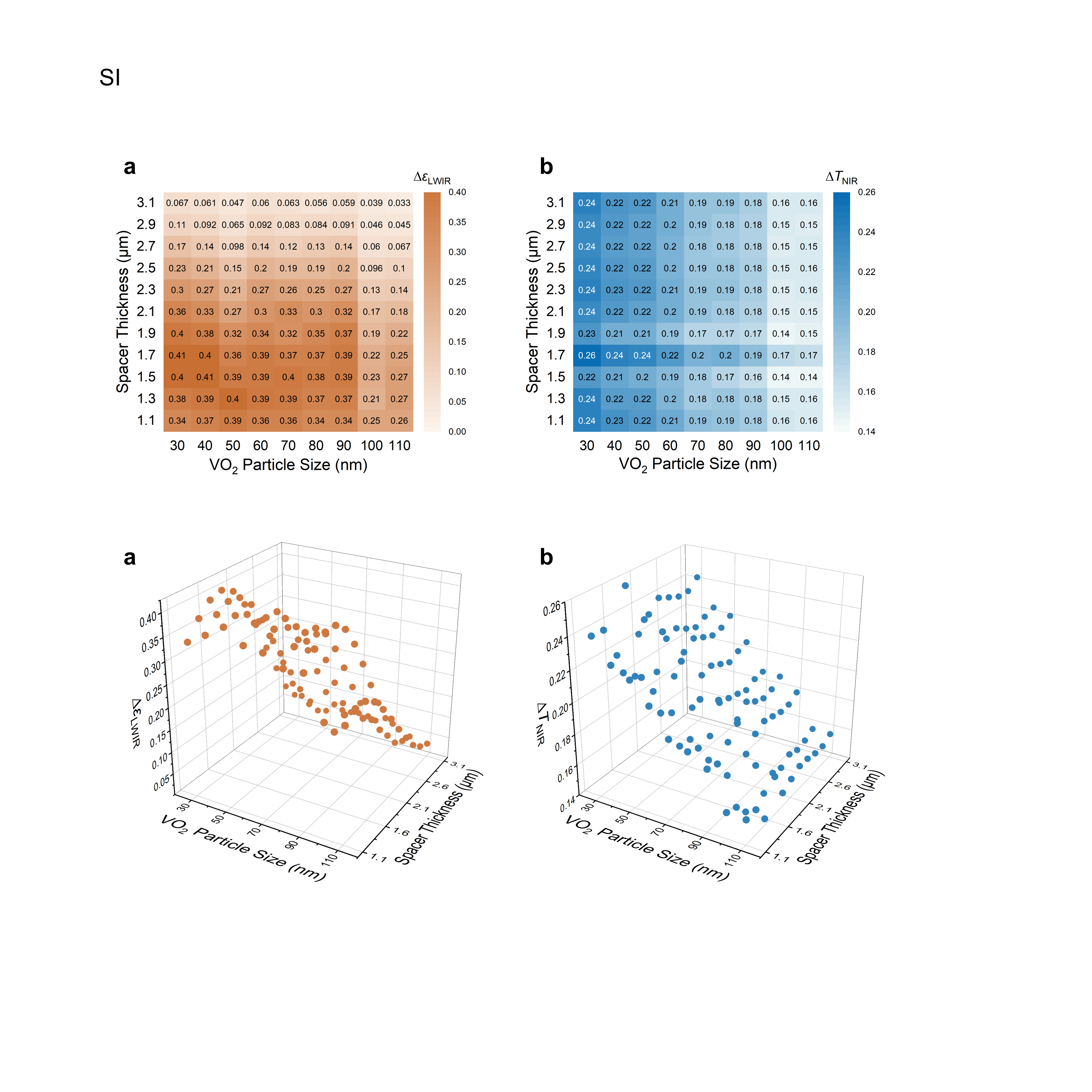


Figure S. Three-dimensional distribution of the raw simulated optical performance. a, 3D surface plot illustrating the variation of Δ*ε*_LWIR_ with VO_2_ diameter and spacer thickness. b, 3D surface plot illustrating Δ*T*_NIR_ with the two structural parameters.


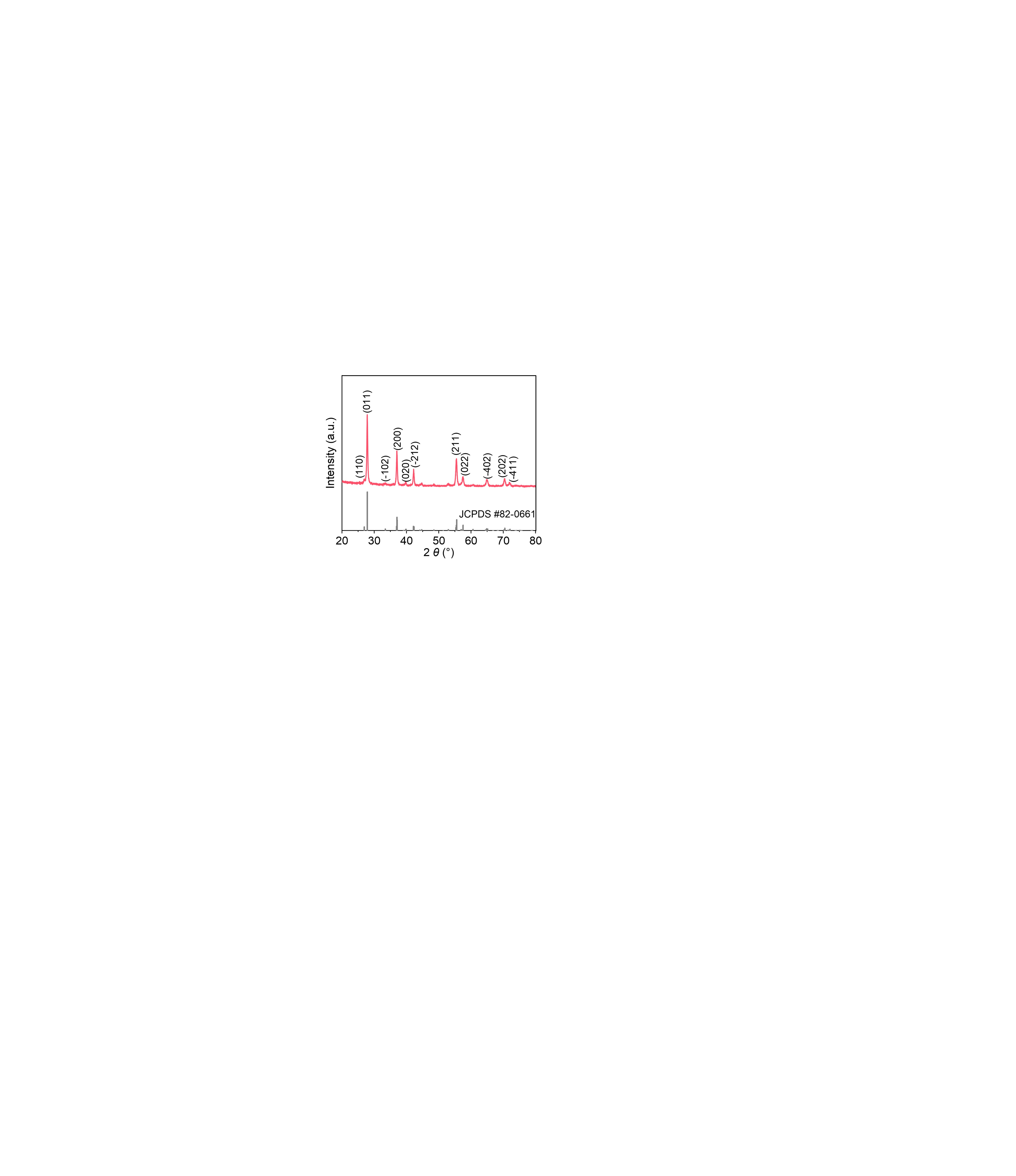


Figure S. XRD pattern of VO_2_ particles synthesized in this work.


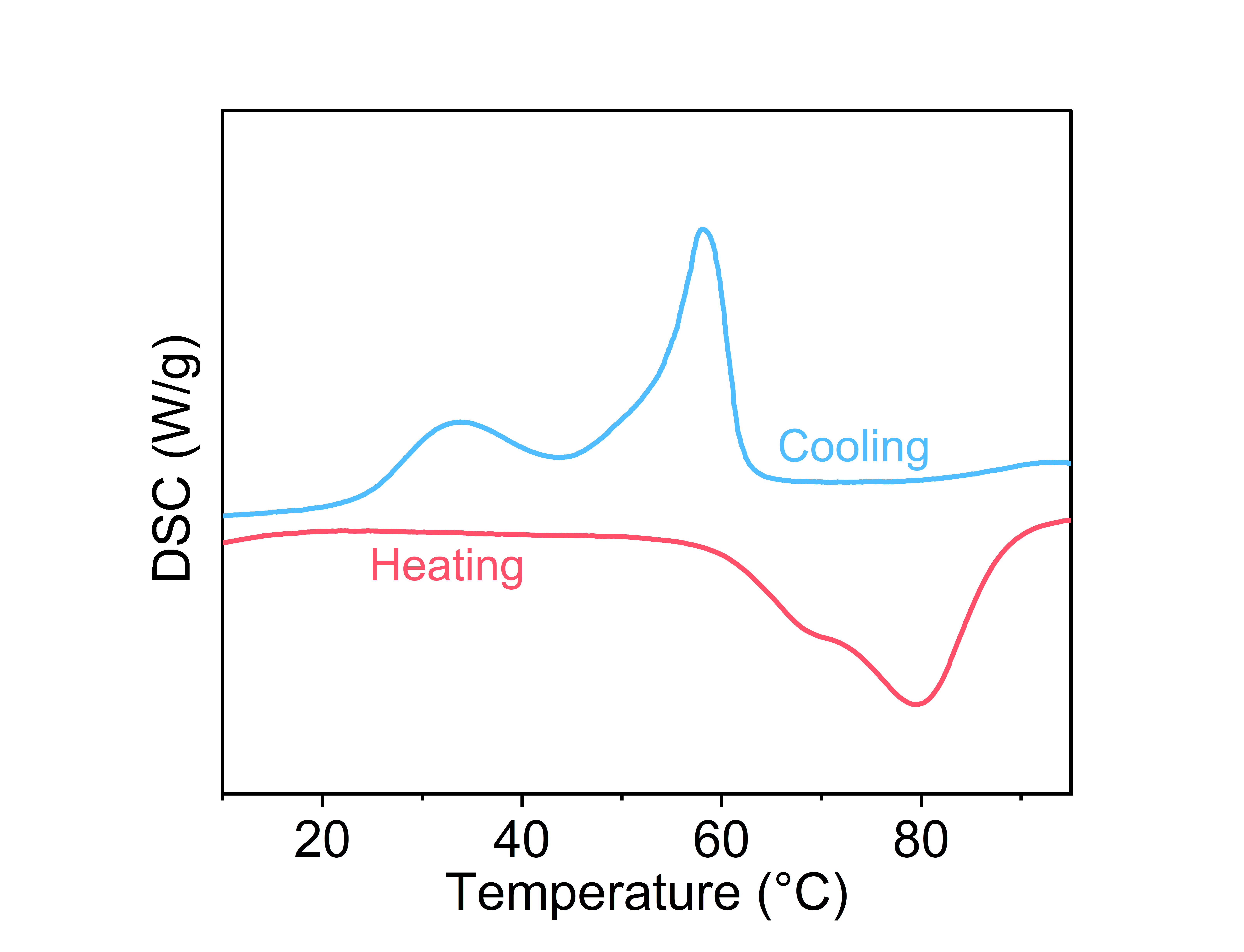


Figure S. DSC curve of the prepared VO_2_ in this work.


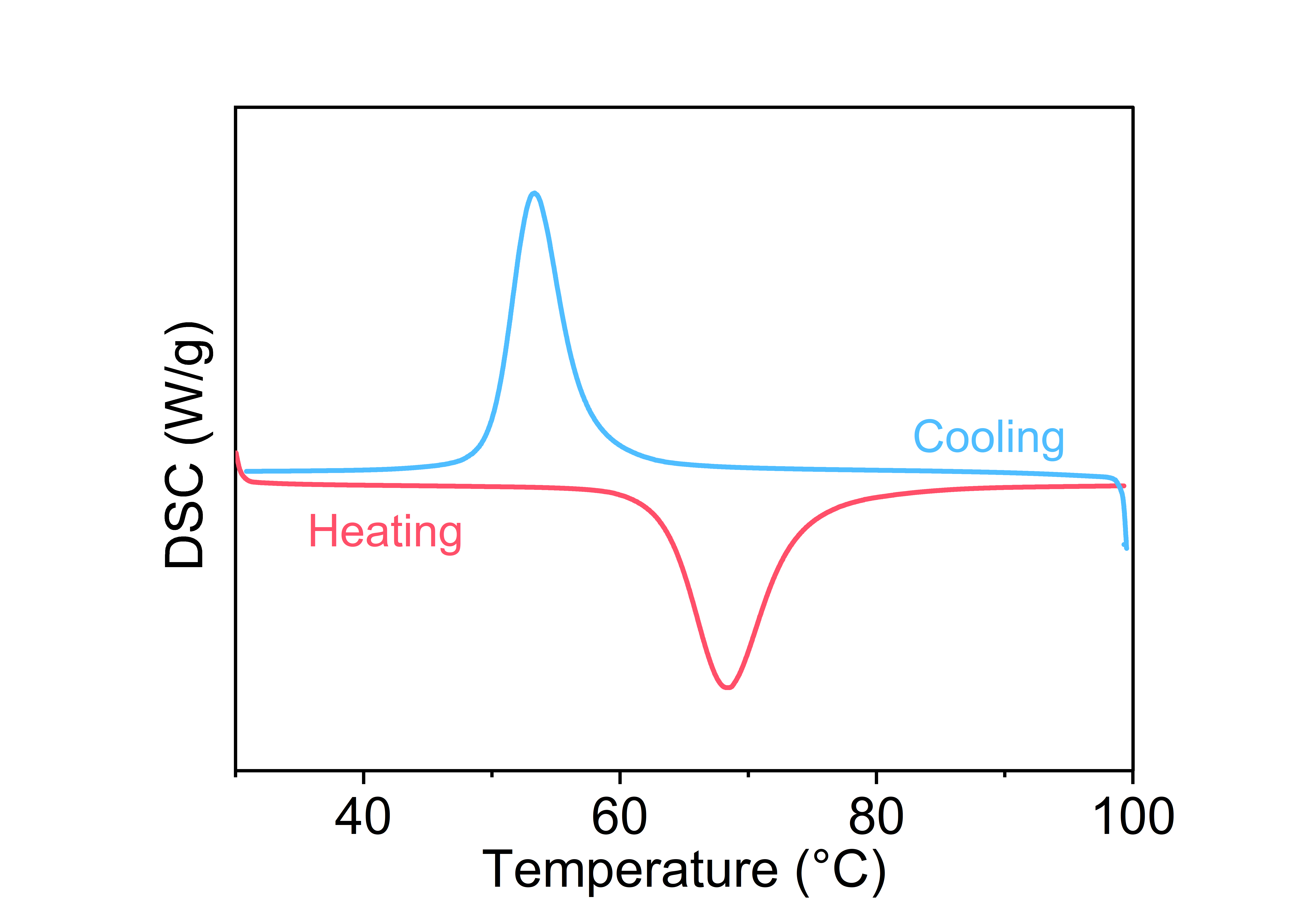


Figure S. DSC curve of the commercial VO_2_.


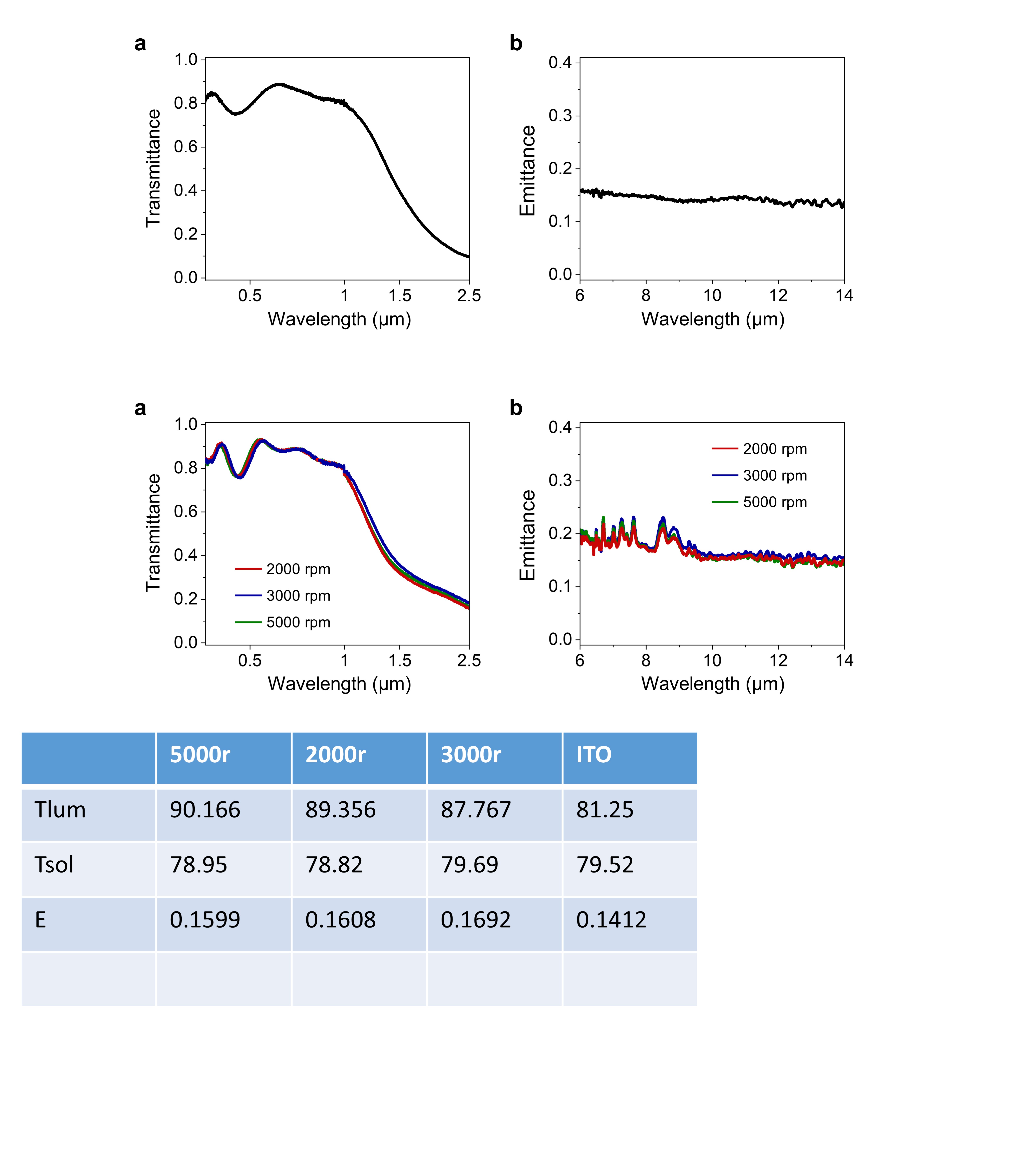


Figure S. a, Solar transmittance of ITO glass with a sheet resistance of 10 Ω/sq. b, Emittance of an ITO glass with a sheet resistance of 10 Ω/sq.


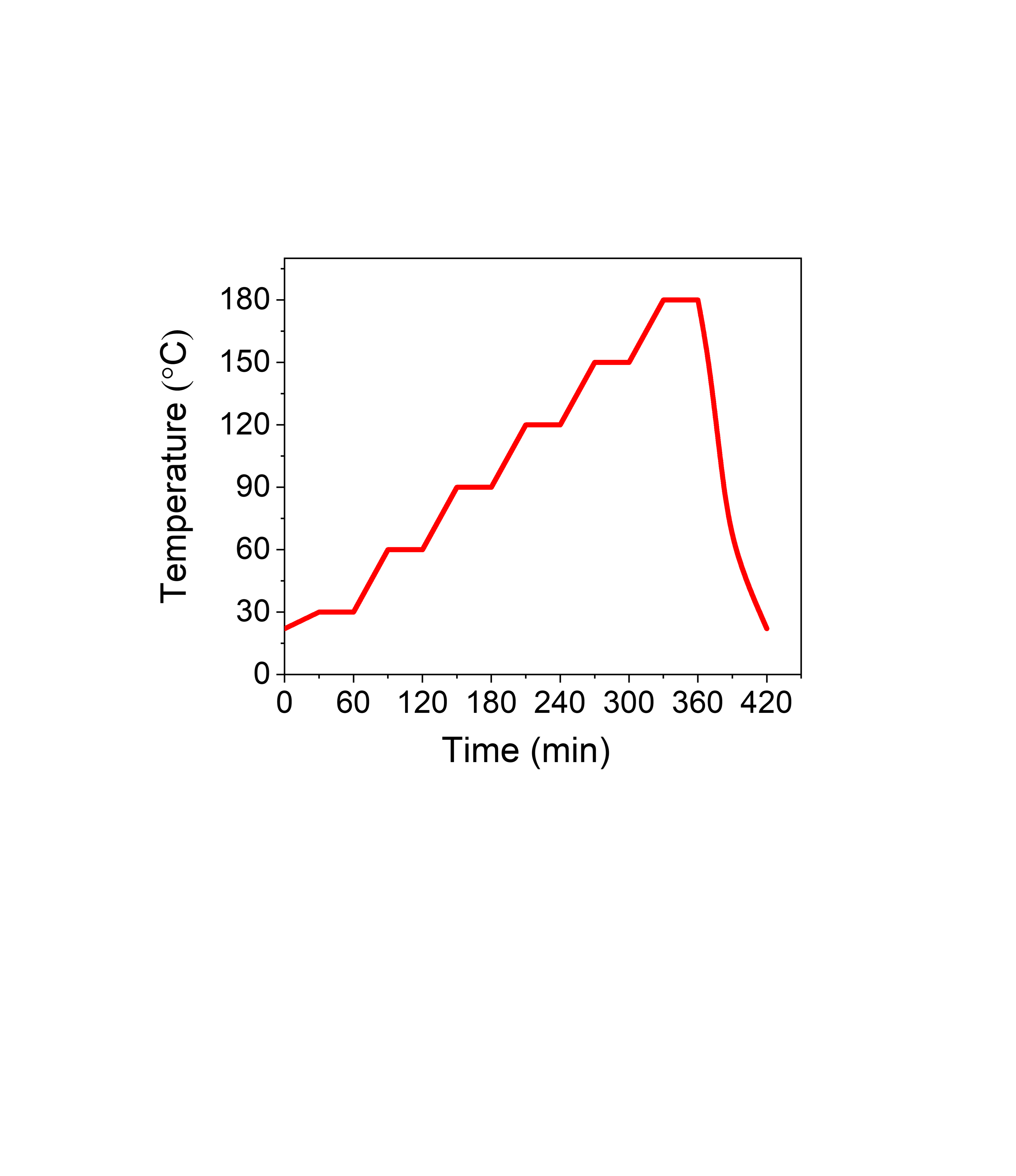


Figure S. Gradient curing process of PI spacer with varying oven temperatures.


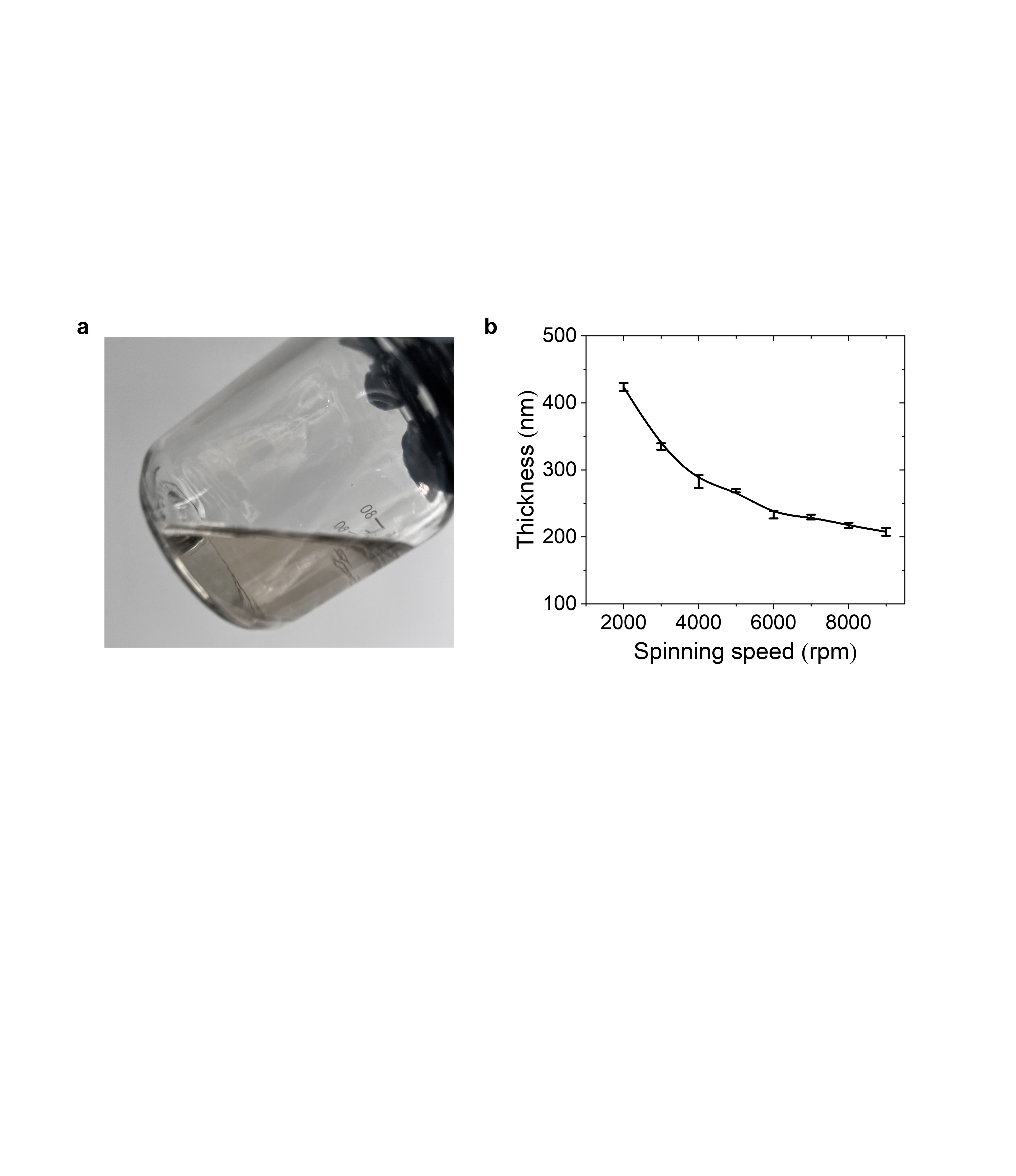


Figure S. a, Polyimide (PI) solution prepared for spacer coating. b, Thickness of the spin-coated PI spacer as a function of spin speed after curing.


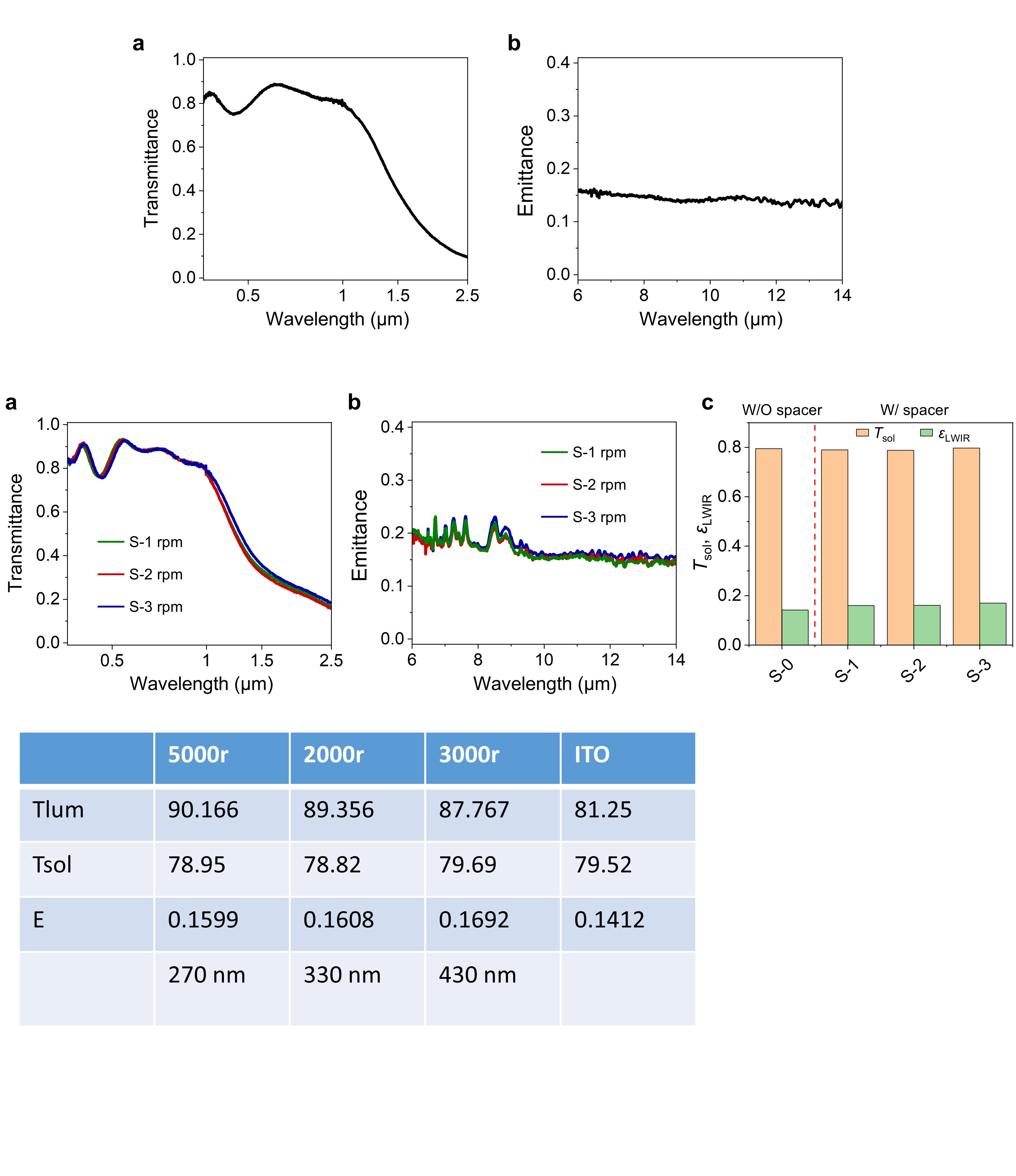


Figure S. a, Solar transmittance of the coated ITO glass with different spacer thickness. b, LWIR emittance of the coated ITO glass with different spacer thickness. c, Calculated *T*_sol_ and *ε*_LWIR_ of bare ITO glass with and without a spacer layer. S-1, S-2 and S-3 denote spacer thicknesses of 270, 335 and 423 nm, respectively.


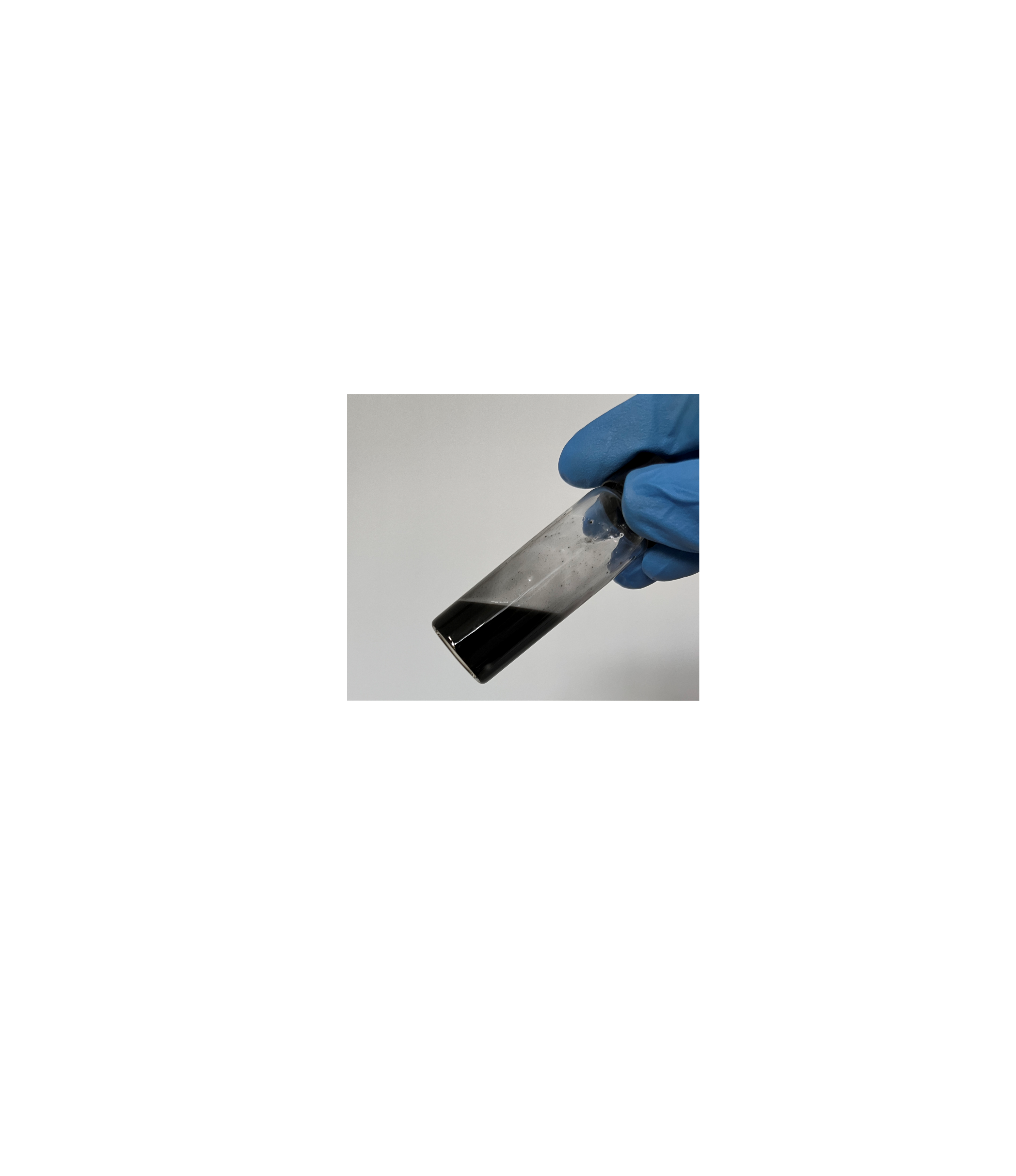


Figure S. VO_2_ nanoparticle inks prepared for spin-coating.


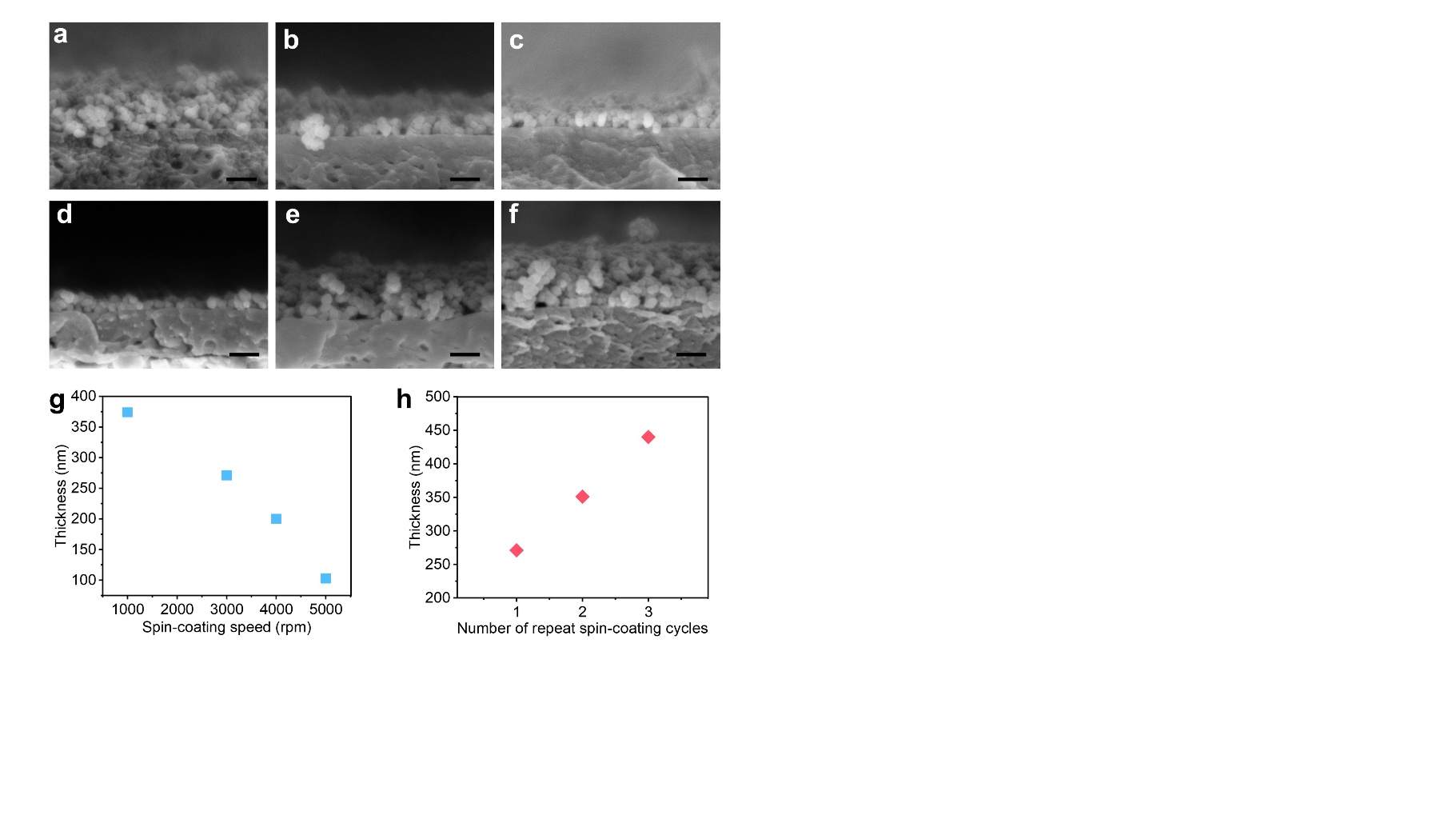


Figure S. Cross-section SEM images of films with the top VO_2_ layer fabricating by different spin-coating processes: (a) 1000 rpm for 60 seconds, (b) 3000 rpm for 60 seconds, (c) 4000 rpm for 60 seconds, (d) 5000 rpm for 60 seconds, (e) 3000 rpm for 60 seconds, repeated twice, and (f) 3000 rpm for 60 s, repeated three times. (g) The relationship between the thickness of VO_2_ layer and the spin-coating speed. (h) The relationship between the thickness of VO_2_ layer and the number of repeat spin-coating cycles at 3000 rpm for 60 seconds. Scale bar: 200 nm.


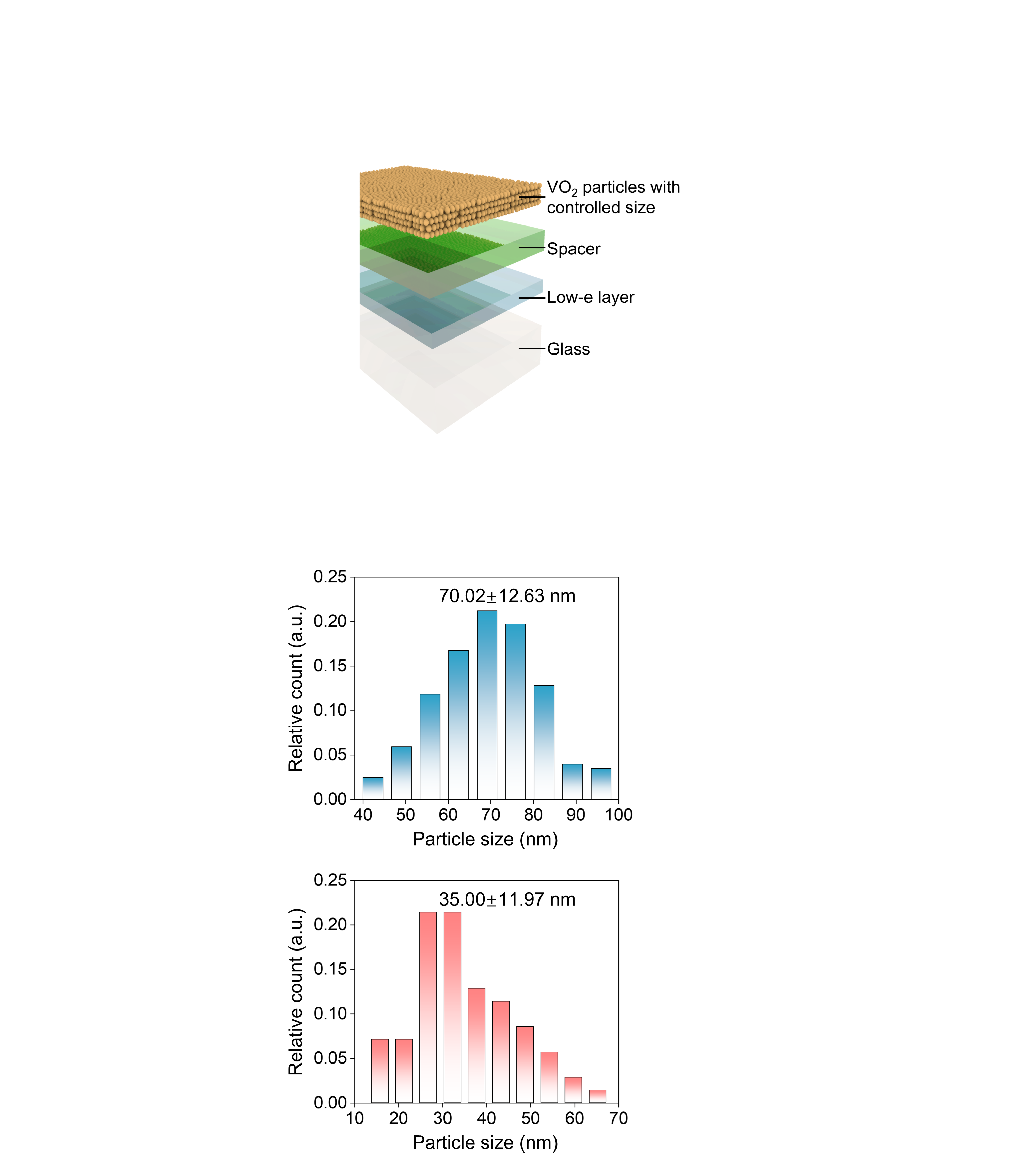


Figure S. Particle-size distribution of conventional large‑diameter VO_2_ particles.


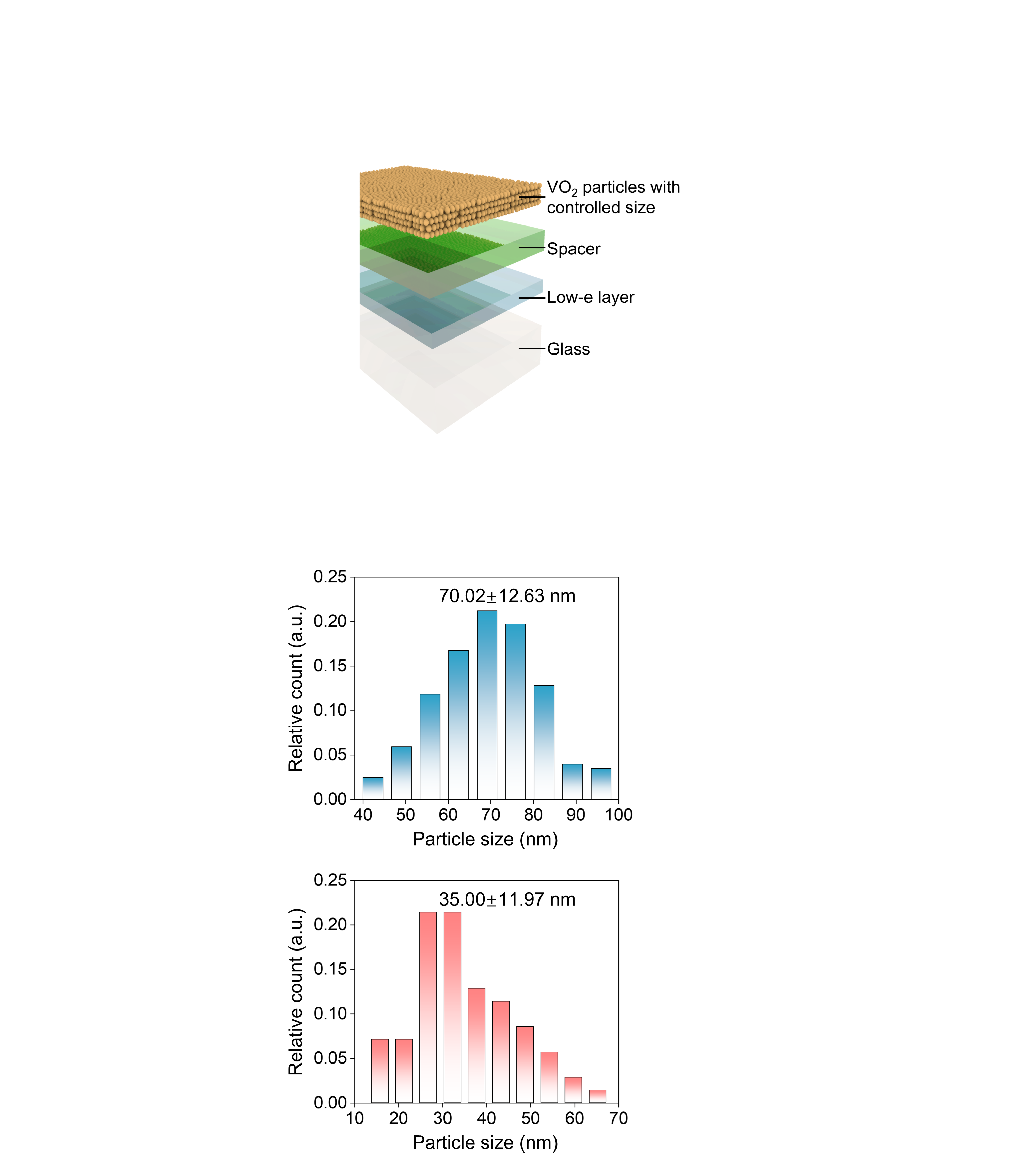


Figure S. Particle-size distribution of our synthesized, size-controlled VO_2_ particles.


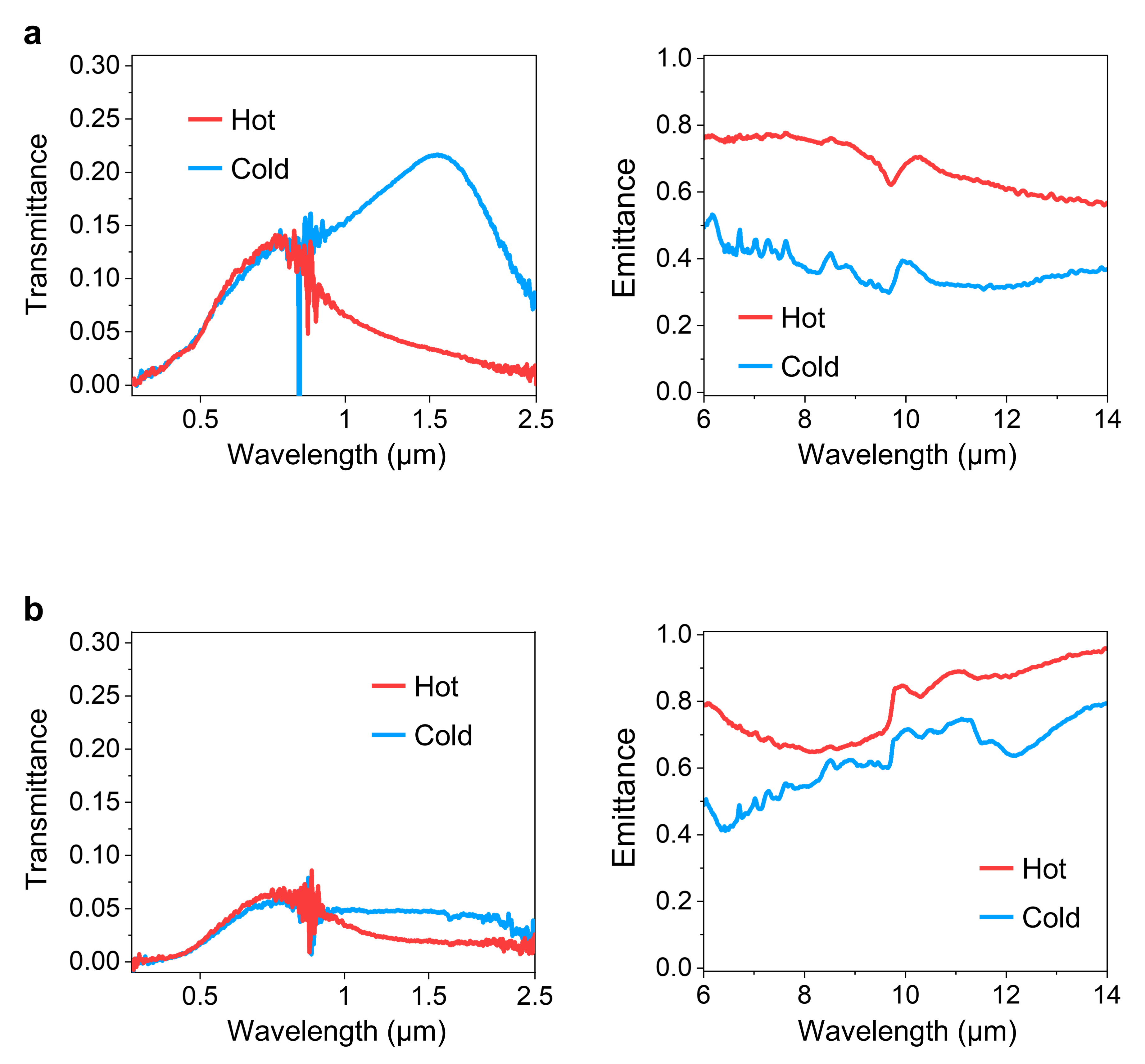


Figure S. a, Solar transmittance (left) and thermal emittance (right) of the smart window incorporating size-controlled VO_2_ particles with a 270 nm spacer layer. b, Solar transmittance (left) and thermal emittance (right) of the smart window incorporating large VO_2_ particles with a 270 nm spacer layer.


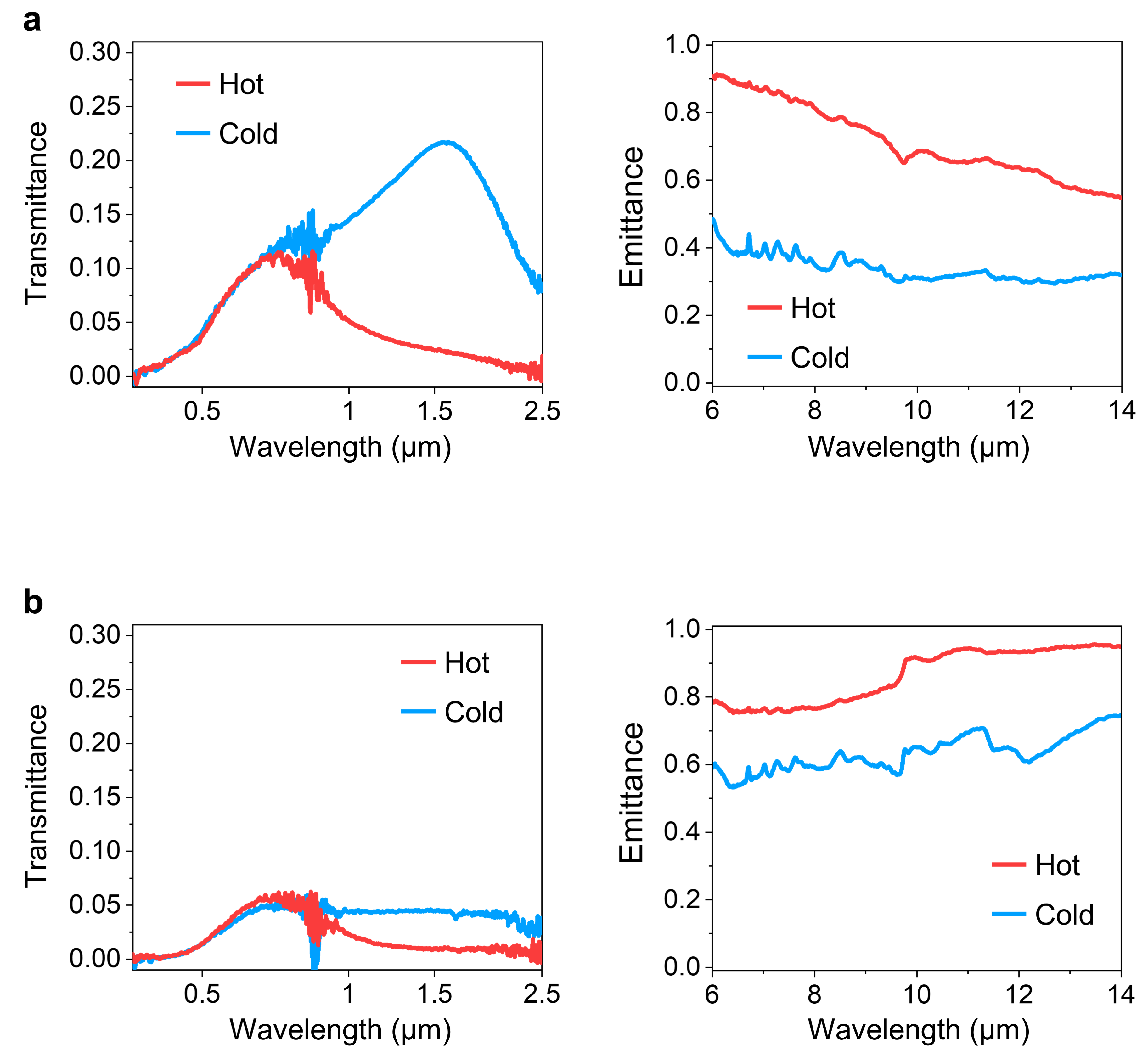


Figure S. a, Solar transmittance (left) and thermal emittance (right) of the smart window incorporating size-controlled VO_2_ particles with a 335 nm spacer layer. b, Solar transmittance (left) and thermal emittance (right) of the smart window incorporating large VO_2_ particles with a 335 nm spacer layer.


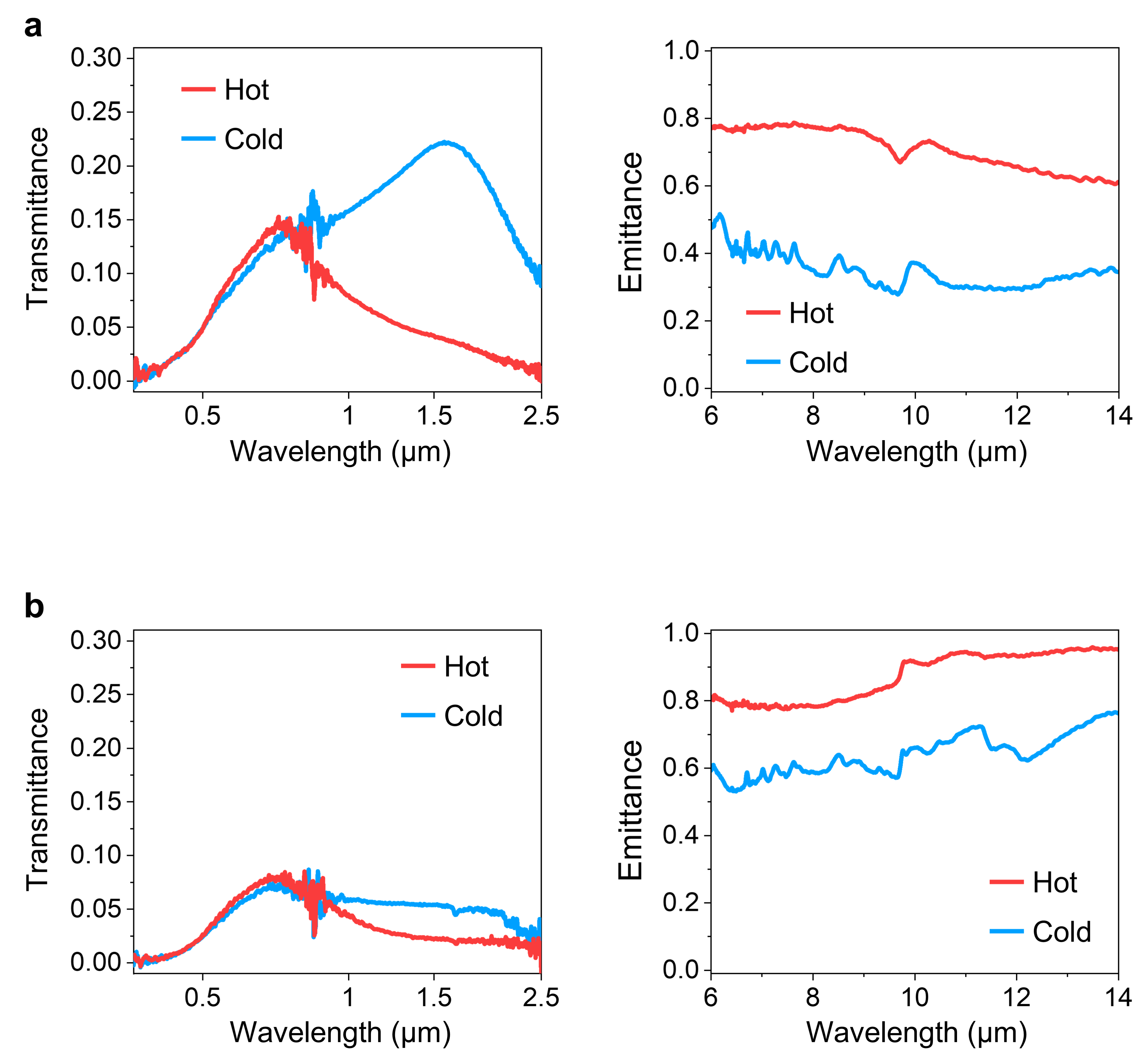


Figure S. a, Solar transmittance (left) and thermal emittance (right) of the smart window incorporating size-controlled VO_2_ particles with a 423 nm spacer layer. b, Solar transmittance (left) and thermal emittance (right) of the smart window incorporating large VO_2_ particles with a 423 nm spacer layer.


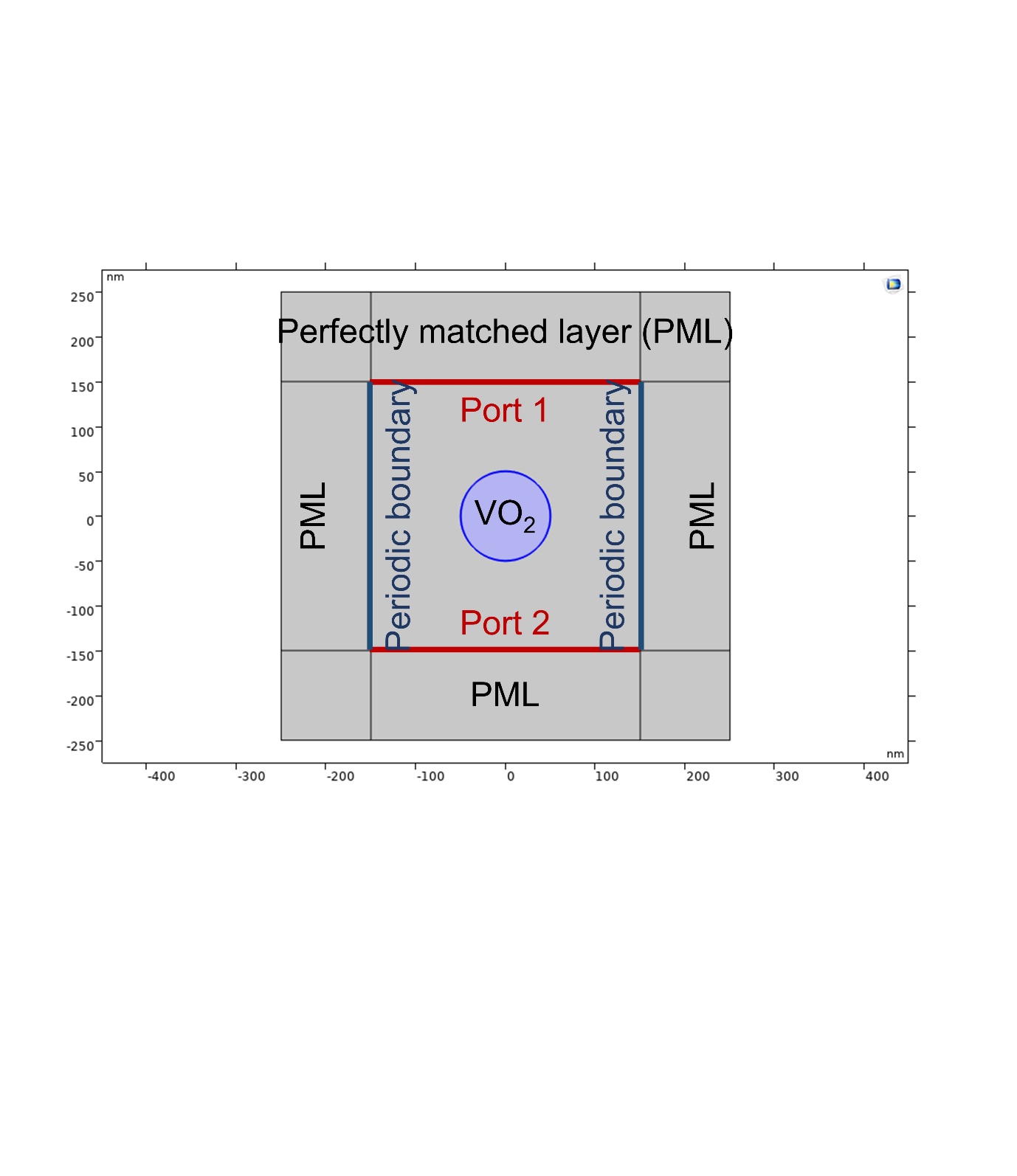


Figure S. The optical simulation model.


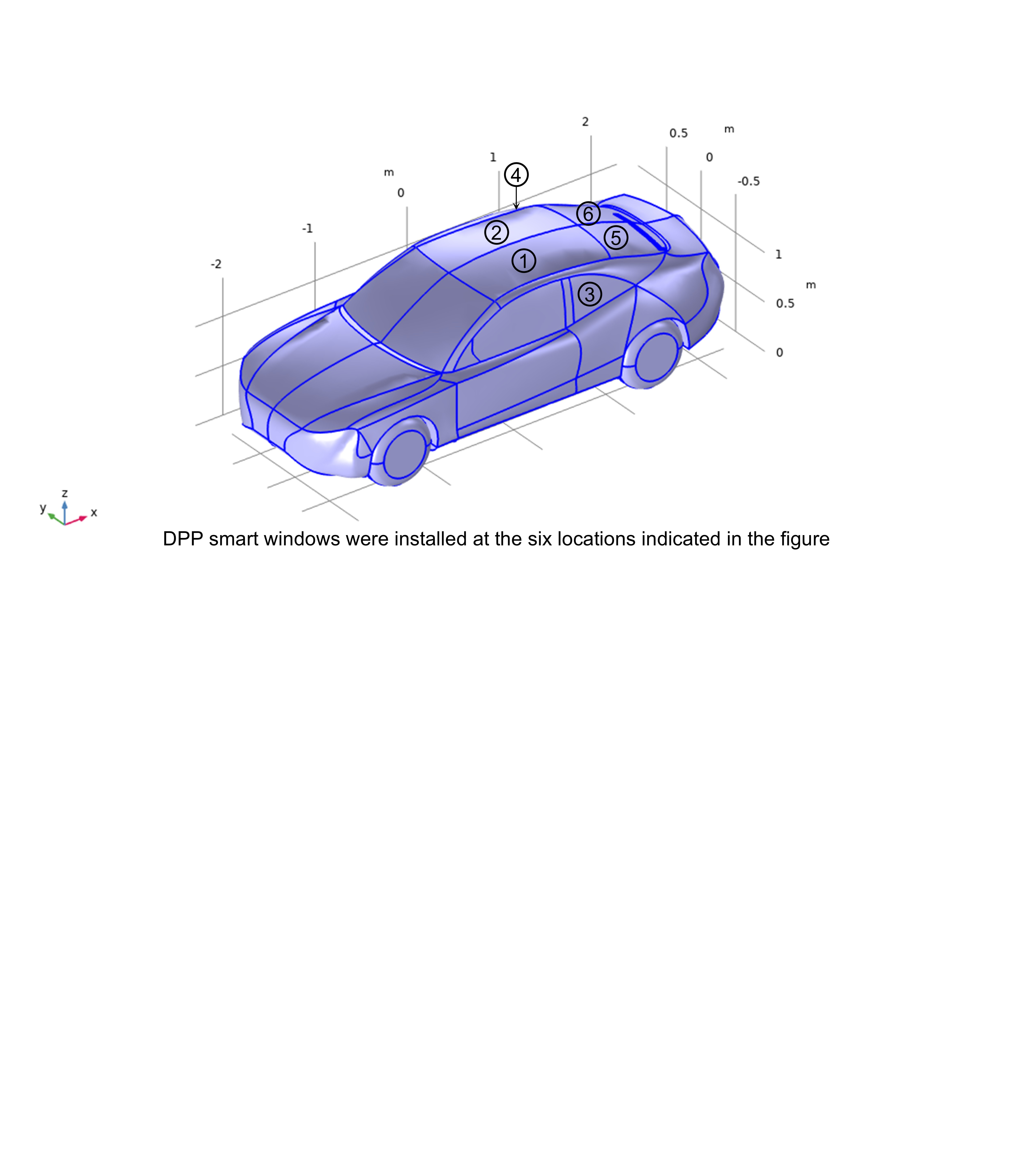


Figure S. The car model used in the heat-transfer simulation.


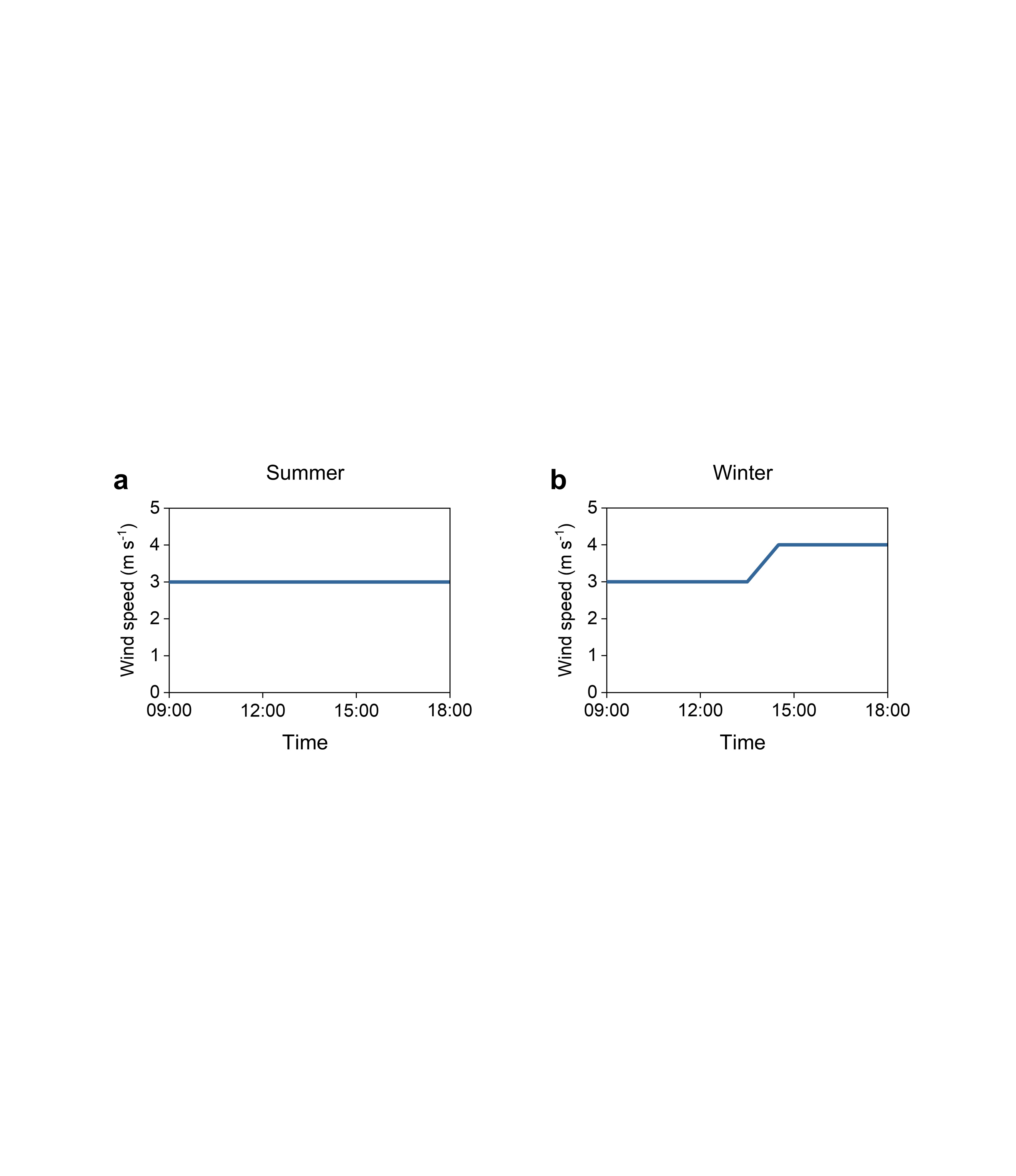


Figure S. Wind speed for Beijing, China (39.8° N, 116.5° E) during (a) a typical summer day (July 21st) and (b) a typical winter day (January 1st).


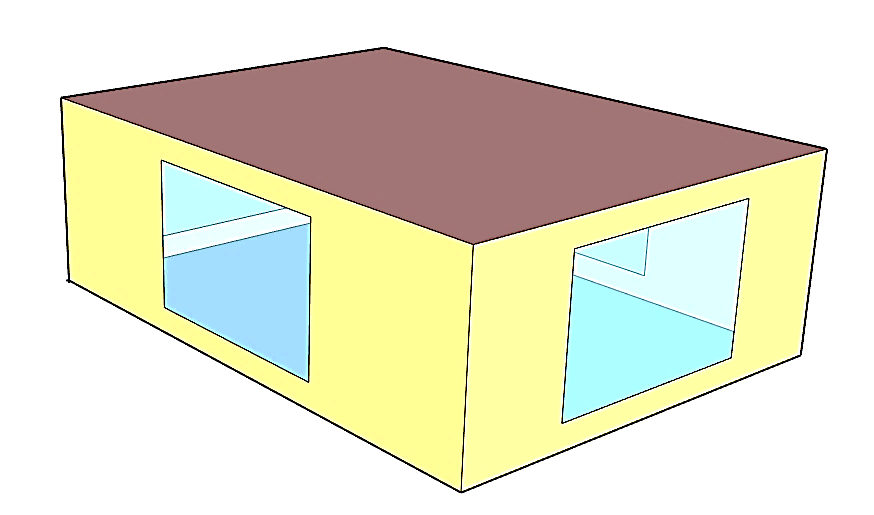


Figure S. The building model used in the energy-saving simulation, which has a window area of 24 m^2^, and a gross window-wall ratio of 31.75%.


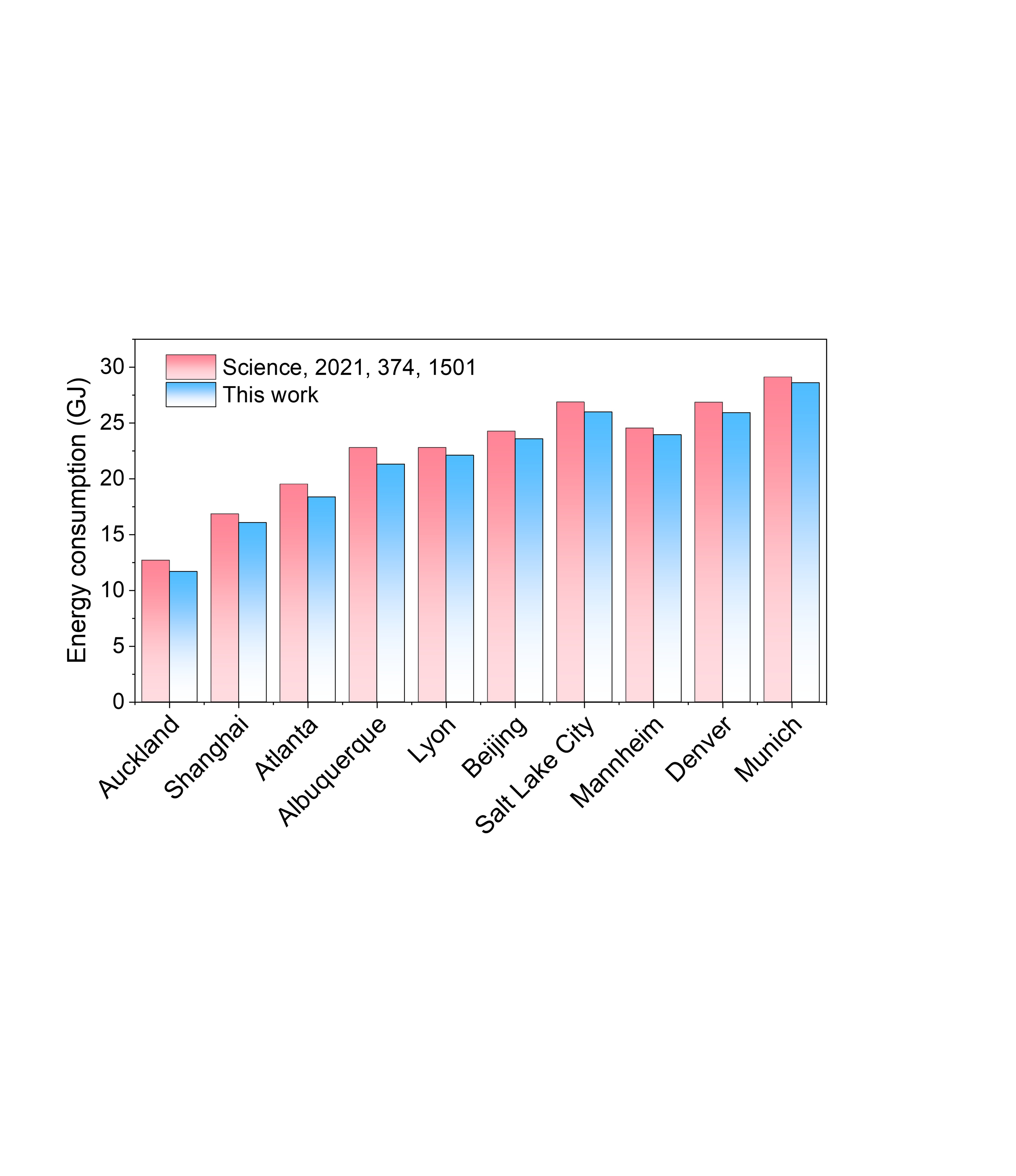


Figure S. Comparison of the simulated annual building energy consumption between the proposed DPP smart window and a state-of-the-art thermochromic device (Science, 2021, 374, 1501)^2^.

Supplementary Table 1. Comparisons with other smart window systems.

|  | Electrochromic/ mechanochromic smart windows | Thermochromic smart windows (this work) |
| --- | --- | --- |
| Actuation | Active | Passive |
| Device complexity degree | Complex, control system is required | Simple, using simple coating or encapsulation |
| For energy-saving windows | Suitable | Suitable |
| Optical transition speed | Acceptable | Acceptable |

Supplementary Table 2. Examples of the cutting-edge VO_2_-based smart windows reported in the literature.

| Material/structure | Method | NIR spectra | LWIR/Broadband spectra | Reference |
| --- | --- | --- | --- | --- |
| VO_2_/PMMA/ITO | Trial-and-error optimization | Δ*T*_NIR_ = 10.2% | Δ*ε*_LWIR_ = 0.4 | Wang et al. Science, 2021, 374, 1501.^2^ |
| VO_2_/PMMA/IHO | Trial-and-error optimization | Δ*T*_NIR_ = 10.9% | Δ*ε*_LWIR_ = 0.26 | Li et al. Nano energy, 2024, 129, 110023.^3^ |
| VO_2_/PMMA/AgNWs | Trial-and-error optimization | Δ*T*_NIR_ < 2% | Δ*ε*_LWIR_ = 0.44 | Hu et al. Nano Lett., 2024, 24, 657.^4^ |
| VO_2_/PAN/AgNWs | Trial-and-error optimization | NA | Δ*ε*_LWIR_ = 0.5 | Li et al. J. Materiomics, 2025, 11, 100871.^5^ |
| Size-controlled VO_2_/PI spacer/ITO | Physics-guided neural network | Δ*T*_NIR_ = 12% | Δ*ε*_LWIR_ = 0.56 | This work |

Supplementary Table 3. Details of the used commercial glass.

|  | **Manufacturer name** | ***T*_lum_** | ***T*_NIR_** | ***ε*_LWIR_** | **Number** |
| --- | --- | --- | --- | --- | --- |
| C1 | Vitro | 0.09 | 0.07 | 0.394 | 450 |
| C2 | Vitro | 0.091 | 0.064 | 0.293 | 452 |
| C3 | Saint-Gobain Glass | 0.091 | 0.081 | 0.68 | 21294 |
| C4 | Glas Trösch AG | 0.094 | 0.072 | 0.458 | 14108 |
| C5 | Pilkington North America | 0.096 | 0.34 | 0.846 | 9988 |
| C6 | las Trösch AG | 0.098 | 0.082 | 0.458 | 14098 |
| C7 | Glas Trösch AG | 0.103 | 0.07 | 0.292 | 14930 |
| C8 | Glas Trösch AG | 0.103 | 0.075 | 0.292 | 14938 |
| C9 | Glas Trösch AG | 0.104 | 0.079 | 0.292 | 14936 |
|  | **Manufacturer name** | ***T*_lum_** | ***T*_sol_** | ***ε*_LWIR_** | **Number** |
| L1 | Glas Trösch AG | 0.098 | 0.061 | 0.051 | 14270 |
| L2 | Guardian Middle East | 0.2 | 0.194 | 0.577 | 12199 |
| L3 | Pilkington North America | 0.3 | 0.155 | 0.167 | 9956 |
| L4 | Vitro formerly PPG | 0.4 | 0.145 | 0.018 | 5392 |
| L5 | XYG Glass | 0.5 | 0.236 | 0.054 | 2961 |
| L6 | Taiwan Glass Ind. Corp. | 0.6 | 0.226 | 0.033 | 6521 |
| L7 | AGC Glass Co. N.A. | 0.7 | 0.444 | 0.047 | 1043 |
| L8 | China Southern Glass | 0.8 | 0.57 | 0.13 | 1631 |
| L9 | Saint-Gobain Glass | 0.9 | 0.642 | 0.05 | 21443 |

Supplementary Table 4. Comparisons with other ML-based studies.

| Main strategy | Optical properties | Application | Reference |
| --- | --- | --- | --- |
| ML-based paradigm for ultrabroadband and band-selective thermal meta-emitters | Solar reflection and LWIR emission | Passive daytime radiative cooling | Xiao et al., Nature, 2025, 643, 80.^6^ |
| A synergistic genetic algorithm and machine learning strategy for photonic design | Selective transmission of solar radiation | A coolhouse film for plant photosynthesis | Li et al., Nat. Commun., 2025, 16, 1396.^7^ |
| A mixed-integer memetic algorithm for the top emitter and a tandem neural network for the color generation | Solar transmission and LWIR emission | Passive daytime radiative cooling | Guan et al., ACS Photonics, 2023, 10, 715.^8^ |
| A bidirectional neural network for forward prediction of the optical properties of energy-saving windows | Visible transmission and NIR reflection | Energy-saving windows | Wang et al., Photonics Nanostruct. Fundam. Appl., 2025, 65, 101389.^9^ |
| An artificial neural network was trained to achieve the optimal performance (*T*_lum_ and Δ*T*_sol_) | Visible transmission and solar modulation | Smart windows | Balin et al., Opt. Express, 2019, 27, A1030.^10^ |
| A physics-guided neural network for the inverse design of thermochromic smart windows | Solar and LWIR modulation | FP smart windows | This work |

Supplementary Table 5. Quantitative comparison of computational time complexity.

| **Methods** | **Time per Single Evaluation** | **Time for Inverse Design**  **(5000 evaluations)** | **Computational Platform & Mesh Conditions** |
| --- | --- | --- | --- |
| 3D FDTD Simulation | ~ 35 minutes | ~ 122 days | Workstation (Intel Xeon w7-2495X CPU) |
| Our Surrogate | < 0.1 milliseconds | ~ 30 seconds | Personal Computer (Intel Core i5-13400 CPU) |

Supplementary Table 6. Definition of conditions in FDTD simulations.

| VO_2_ particle | | Spacer | |
| --- | --- | --- | --- |
| Size (nm) | Layer (n) | Thickness (μm) | Step size (μm) |
| 30 | 3 | 1.1 ~ 3.1 | 0.2 |
| 40 | 3 | 1.1 ~ 3.1 | 0.2 |
| 50 | 2 | 1.1 ~ 3.1 | 0.2 |
| 60 | 2 | 1.1 ~ 3.1 | 0.2 |
| 70 | 2 | 1.1 ~ 3.1 | 0.2 |
| 80 | 1 | 1.1 ~ 3.1 | 0.2 |
| 90 | 1 | 1.1 ~ 3.1 | 0.2 |
| 100 | 1 | 1.1 ~ 3.1 | 0.2 |
| 110 | 1 | 1.1 ~ 3.1 | 0.2 |

Supplementary Table 7. Comparison of privacy-protection performance, Δ*T*_NIR_, and Δ*ε*_LWIR_ among the present work, commercial glass, and reported results.

|  | This work | Science, 2021, 374, 1501.^2^ | Commercial privacy glass |
| --- | --- | --- | --- |
| Privacy protection  ability  =(1-*T*_lum_)/0.9 | 100% | 80% | 100% |
| Δ*T*_NIR_ | 12% | 10% | 0 |
| Δ*ε*_LWIR_ | 0.56 | 0.4 | 0 |

Supplementary Table 8. Global definitions for heat‑transfer simulations.

| Items | | Value | Unit |
| --- | --- | --- | --- |
| Road surface | Thermal conductivity | 0.3 | W/(m K) |
|  | Density | 1300 | kg/m^3^ |
|  | Isobaric heat capacity | 800 | J/(kg K) |
|  | Solar absorptance | 0.9 | 1 |
|  | Thermal emittance | 0.95 | 1 |
| Electrical vehicle | Relative permeability | 1 | 1 |
|  | Isobaric heat capacity | 900 | J/(kg K) |
|  | Thermal conductivity | 238 | W/(m K) |
|  | Density | 2700 | kg/m^3^ |
|  | Tire emissivity | 0.9 | 1 |
|  | Metallic paint emissivity | 0.5 | 1 |
|  | Metallic paint solar absorptance | 0.5 | 1 |
| Front car glass | Relative permeability | 1 | 1 |
|  | Density | 2210 | kg/m^3^ |
|  | Thermal conductivity | 1.4 | W/(m K) |
|  | Isobaric heat capacity | 730 | J/(kg K) |
|  | Solar transmittance | 0.9 | 1 |
|  | Thermal emittance | 0.84 | 1 |
| Commercial privacy glass (C1) | Solar transmittance | 0.07 | 1 |
|  | Thermal emittance | 0.394 | 1 |
| Summer | Solar irradiance | 1000 | W m^-2^ |
|  | Ambient temperature | 30 | ℃ |
|  | Heat transfer coefficient | 10 | W/(m^2^ K) |
| Winter | Solar irradiance | 300 | W m^-2^ |
|  | Ambient temperature | 0 | ℃ |
|  | Heat transfer coefficient | 10 | W/(m^2^ K) |

Supplementary Table 9. Information of the building model.

| Items | Specifications |
| --- | --- |
| Window fraction (window-to-wall ratio) | 31.75% of above-grade gross walls |
| Window locations | Even distribution among all four sides |
| Exterior walls | Stucco + concrete + wall insulation + gypsum |
| Roof | Roof membrane + roof insulation + metal decking |
| Interior design temperatures | 20°C (winter)/24°C (summer) |

Supplementary Table 10. The representative cities in mid-latitude regions.

| Cities | Latitude | Longitude | Climate zone |
| --- | --- | --- | --- |
| Auckland, New Zealand | -37.009 | 174.807 | 3A |
| Shanghai, China | 31.391 | 121.445 | 3A |
| Atlanta, GA, U.S. | 33.63 | -84.442 | 3A |
| Albuquerque | 35.038 | -106.622 | 4B |
| Lyon, France | 45.726 | 5.078 | 4A |
| Beijing, China | 39.806 | 116.469 | 4A |
| Salt Lake City, UT, U.S. | 40.778 | -111.969 | 4B |
| Mannheim, Germany | 49.49 | 8.47 | 5A |
| Denver, CO, U.S. | 39.767 | -104.869 | 5B |
| Munich, Germany | 48.348 | 11.813 | 5A |

Supplementary Table 11. Optical properties for different windows used in simulation.

| Optical properties | Commercial privacy glass (C5) | Low-e  privacy glass  (C6) | This work  (cold state) | This work  (hot state) |
| --- | --- | --- | --- | --- |
| *T*_sol_ | 0.242 | 0.092 | 0.124 | 0.062 |
| *R*_sol-f_ | 0.361 | 0.337 | 0.108 | 0.088 |
| *R*_sol-b_ | 0.117 | 0.1 | 0.14 | 0.156 |
| *T*_lum_ | 0.096 | 0.098 | 0.095 | 0.075 |
| *R*_lum-f_ | 0.747 | 0.281 | 0.071 | 0.064 |
| *R*_lum-b_ | 0.191 | 0.1 | 0.085 | 0.104 |
| *ε* | 0.846 | 0.458 | 0.32 | 0.77 |

Reference

1 Polyanskiy, M. N. *Refractive index database*, <<https://refractiveindex.info/>> (2017).

2 Wang, S. C. et al*.* Scalable thermochromic smart windows with passive radiative cooling regulation. *Science* **374**, 1501-1504 (2021).

3 Li, S. J. et al. Self-adaptive energy-efficient windows with enhanced synergistic regulation of broadband infrared thermal radiation. *Nano Energy* **129**, 110023 (2024).

4 Hu, X. et al. Facile and widely applicable route to self-adaptive emissivity modulation: energy-saving demonstration with transparent wood. *Nano Letters* **24**, 657-666 (2024).

5 Li, J. et al. Fabry–pérot cavity smart windows with superior solar and thermal modulation capabilities. Journal of Materiomics **11**, 100871 (2025).

6 Xiao, C. Y. et al. Ultrabroadband and band-selective thermal meta-emitters by machine learning. *Nature* **643**, 80-88 (2025).

7 Li, J. L. et al. Accelerated photonic design of coolhouse film for photosynthesis via machine learning. Nature Communications **16**, 1396 (2025).

8 Guan, Q. S. et al. Machine learning-enabled inverse design of radiative cooling film with on-demand transmissive color. *ACS Photonics* **10**, 715-726 (2023).

9 Wang, C. C. et al. Machine-learning-assisted design of energy-saving windows with high near-infrared shielding properties. *Photonics and Nanostructures - Fundamentals and Applications* **65**, 101389 (2025).

10 Balin, I. et al. Training artificial neural network for optimization of nanostructured VO_2_-based smart window performance. *Optics Express* **27**, A1030-A1040 (2019).
